# Metabolic inflammation, brain age and cognitive functioning in short- and long-term clinical weight loss trials

**DOI:** 10.1016/j.ebiom.2025.106064

**Published:** 2025-12-02

**Authors:** Lukas Maurer, Leonard Kozarzewski, Linus Haberbosch, Agnes Flöel, John-Dylan Haynes, Joachim Spranger, Knut Mai, Martin Weygandt

**Affiliations:** a Department of Endocrinology and Metabolism, Charité – Universitätsmedizin Berlin, Corporate Member of Freie Universität Berlin, Humboldt-Universität zu Berlin, and Berlin Institute of Health, European Reference Network on Rare Endocrine Diseases (ENDO-ERN), 10117, Berlin, Germany; b Pituitary Tumor Center of Excellence at Charité – Universitätsmedizin Berlin, Corporate Member of Freie Universität Berlin, Humboldt-Universität zu Berlin, and Berlin Institute of Health, Charitéplatz 1, 10117, Berlin, Germany; c Cambridge Endocrine Molecular Imaging Group, Institute of Metabolic Science, University of Cambridge, National Institute for Health Research Cambridge Biomedical Research Centre, Addenbrooke's Hospital, Hills Road, Cambridge, CB2 0QQ, UK; d Department of Neurology, University Medicine Greifswald, Greifswald, 17475, Germany; e Experimental and Clinical Research Center, A Cooperation between the Max Delbrück Center for Molecular Medicine in the Helmholtz Association and Charité Universitätsmedizin Berlin, Berlin, Germany; f Charité—Universitätsmedizin Berlin, Corporate Member of Freie Universität Berlin and Humboldt-Universität zu Berlin, Experimental and Clinical Research Center, Lindenberger Weg 80, 13125, Berlin, Germany; g Max Delbrück Center for Molecular Medicine in the Helmholtz Association (MDC), Berlin, Germany; h Cluster of Excellence NeuroCure, Charité – Universitätsmedizin Berlin, Corporate Member of Freie Universität Berlin, Humboldt-Universität zu Berlin, and Berlin Institute of Health, NeuroCure Clinical Research Center, 10117, Berlin, Germany; i Bernstein Center for Computational Neuroscience and Berlin Center for Advanced Neuroimaging and Clinic for Neurology, Charité Universitätsmedizin, Corporate Member of Freie Universität Berlin, Humboldt Universität zu Berlin, and Berlin Institute of Health, Berlin, Philippstraße 13, Haus 6, 10115, Germany; j Department of Human Nutrition, German Institute of Human Nutrition Potsdam-Rehbrücke, Nuthetal, Germany

**Keywords:** Brain health, Metabolic inflammation, Obesity, Brain age, Cognitive functioning

## Abstract

**Background:**

Observational studies suggest that metabolic inflammation in obesity can impair brain health, but studies on beneficial effects of weight loss-induced improvements in such markers on brain health and their consequences for clinical outcomes are scarce.

**Methods:**

Consequently, we investigated 53 obese participants in a short-term dietary weight loss trial (up to 4 months, 137 samples; “Muscle Metabolism Study” or “MMS”) and 30 in an independent long-term trial (up to 39 months, 100 samples; “Maintain”). For each participant and visit, brain health was characterised in terms of the “brain-predicted age difference” (“brain-PAD”; the difference of the age of a person predicted with machine learning from structural brain MRI minus their chronological age). Increasingly positive brain-PAD scores indicate increasingly poorer brain health. Further, we determined the HOMA index, leptin, fetuin B and CRP levels as markers collectively reflecting low-grade inflammation and impaired metabolic signalling. Finally, we evaluated the relevance of these parameters for brain-PAD and the association of brain-PAD alterations for cognition, which was measured in the MMS with neuropsychological tests.

**Findings:**

Weight loss led to improved brain-PAD scores (MMS: t = −2.02, p = 0.023, effect size partial η^2^ (η^2^_p_) = 0.03; Maintain: t = −7.37, p = 4.2·10^−11^, η^2^_p_ = 0.38). According to a False Discovery Rate (FDR) method-corrected threshold (α_FDR_ = 0.05), HOMA index (MMS: t = 2.28, p_FDR_ = 0.024, η^2^_p_ = 0.04; Maintain: t = 2.33, p_FDR_ = 0.023, η^2^_p_ = 0.08), and leptin (MMS: t = 4.43, p_FDR_ = 4.3·10^−5^, η^2^_p_ = 0.14; Maintain: t = 1.91, p_FDR_ = 0.041, η^2^_p_ = 0.06), showed significant positive links to brain-PAD in both trials, fetuin B did so in Maintain (t = 2.57, p_FDR_ = 0.023, η^2^_p_ = 0.11). Brain-PAD variations were associated with a neuropsychological test of psychomotor speed and visual attention (t = 2.32, p_FDR_ = 0.022, η^2^_p_ = 0.05). Application of explainable artificial intelligence methods showed that this link was parallelled by widespread brain age-related tissue alterations in white and grey matter involved in these functions.

**Interpretation:**

Analyses of two independent weight loss trials suggest that weight loss-induced improvements in metabolic-inflammatory markers have beneficial effects on brain-PAD and the latter were associated with enhancements in cognitive functioning, underscoring the potential clinical relevance of metabolic brain age regulation.

**Funding:**

10.13039/501100001659German Research Foundation; German Ministry for Education and Research, 10.13039/501100017268Berlin Institute of Health; 10.13039/100010447German Centre for Cardiovascular Research; 10.13039/100031584German Center for Diabetes Research.

**Research in context**

**Evidence before this study**

Obesity and type 2 diabetes are linked to accelerated brain ageing and increased risk of cognitive decline. Disrupted metabolic signalling and low-grade inflammation are considered key mechanisms. Brain-predicted age difference (brain-PAD), derived from MRI using machine learning, has recently emerged as a highly sensitive and integrative biomarker of brain health that is not only sensitive to future longevity and neurological disease but that can also extract brain health-related information from multiple tissue. To date, however, there is only very limited knowledge on the specific role of metabolic–inflammatory markers for brain health assessed with this versatile measure in weight regulation trials and associated clinical outcomes.

**Added value of this study**

We analysed two independent dietary weight regulation trials in obesity: a short-term intervention in 53 postmenopausal women (137 MRI samples over 4 months) and a long-term programme including 30 participants (100 MRI samples over 39 months) with phases of weight loss, maintenance, and regain. Across both trials, weight loss was linked to reductions in brain-PAD. Insulin resistance (HOMA index) and leptin showed consistent positive associations with brain-PAD, indicating poorer brain health with higher values. Fetuin B showed a positive association with brain-PAD in the short term trial. Cognitive performance was available only in the short-term trial, where improvements in brain-PAD were accompanied by better scores in a test of neuropsychological test of psychomotor speed and visual attention. These findings support the use of brain-PAD as a novel marker for monitoring brain health and associated clinical outcomes in weight loss interventions, and highlight the importance of metabolic and inflammatory pathways.

**Implications of all the available evidence**

Taken together with observational and interventional data, the present findings suggest that intentional weight loss may have beneficial effects on brain health and cognitive functioning. While larger and longer trials are required to substantiate these observations and clarify underlying mechanisms, the results strengthen the evidence that weight loss in patients with obesity can contribute to healthier brain ageing.

## Introduction

Accumulating evidence suggests that metabolic health affects brain health. For example, Tian et al. (2023)^1^ showed in a very large population-based cohort (>100,000 participants) that the metabolic phenotype is a strong predictor of brain health measured by the brain age machine learning method, which is well-suited for this purpose as it is not only sensitive to future longevity and neurological disease^2^^,^^3^ but it can also utilise different types of tissue alteration.^4^ The method characterises brain health by computing the difference between a person's age predicted by an algorithm based on brain MRI parameters (their “brain age”) minus their chronological age. Higher scores in this parameter, which we refer to as “brain-predicted age differences” (“brain-PAD”) from now on, indicate lower brain health. In central obesity, disrupted metabolic signalling–in particular insulin resistance, altered adipokine (e.g., leptin) and hepatokine (e.g., fetuin B) secretion as well as low-grade inflammation (e.g., indicated by heightened CRP levels) are candidate mechanisms for mediating metabolic-inflammatory brain health effects.^5^, ^6^, ^7^ Specifically, a detrimental effect of insulin resistance on brain health is suggested by findings that obesity and type 2 diabetes mellitus (T2DM) are linked to an increased risk of developing cognitive impairment or even dementia^8^, ^9^, ^10^ and that an increase in diabetes-related biomarkers is associated with a heightened risk for cognitive dysfunction and neurodegeneration even in prediabetes.^11^ Consistently, stronger hippocampal atrophy is often seen in diabetic patients as compared to age-matched controls.^12^ Moreover, although higher leptin in mid-life might protect against Alzheimer's Disease in late-life,^13^ leptin levels heightened by obesity were negatively linked to brain volume in 517 individuals at the time of measurement^14^–which is consistent with leptin's role as proinflammatory cytokine^15^ that can enter the brain and modulate neuroinflammatory responses.^16^ In line with cognitive decline in obesity, animal work shows that leptin resistance leads to reduced hippocampal N-methyl-d-aspartate receptor functioning and structural alterations of hippocampus, which in turn impairs spatial learning and memory (see Valladolid-Acebes et al., 2024 for an overview^17^). Liver damage in obesity is another pivotal feature of metabolic dysfunction. Metabolic dysfunction-associated steatotic liver disease is associated with cognitive impairment in humans^18^ and disease progression is linked to altered thalamus metabolism and reduced cerebral brain volume in animal studies.^19^ The hepatokine fetuin B is elevated in liver-steatosis and patients with type 2 diabetes.^20^ It directly induces a proinflammatory response and glucose intolerance.^21^^,^^22^ Thus, together, these studies suggest that metabolic inflammation might exert detrimental effects on brain health and cognition. However, although various individual brain health markers have been studied in response to weight loss (e.g., resting-state functional connectivity, brain reward processing or regional atrophy;^23^, ^24^, ^25^), studies testing whether the highly integrative brain age measure is not only negatively affected by metabolic markers during heightened weight but may also improve together with them if weight is reduced are rare.^26^, ^27^, ^28^ Moreover, a focal investigation of the role of metabolic-inflammatory markers and of their simultaneous impact of brain-PAD alterations on cognition has not yet been performed.

Consequently, we tested associations of body weight modification-induced alterations in metabolic-inflammatory markers, brain health and cognition. The data evaluated were taken from two body weight modification trials, the four-month “Muscle Metabolism Study” (MMS–ClinicalTrials.gov ID: NCT01105143) and the 39-month “Maintain”-trial (ClinicalTrials.gov ID: NCT00850629). The MRI cohorts of the MMS comprised phases of weight loss and maintenance,^29^^,^^30^ as well as additionally weight regain in the Maintain trial.^31^^,^^32^ Brain health was determined in terms of brain-PAD inferred from structural brain MRI. We first conducted preparatory analyses testing effects of body weight modification on body mass index (BMI) and brain-PAD. Further, we tested associations between BMI and brain-PAD, and between BMI and the biomarkers for metabolic inflammation (HOMA index, leptin, fetuin B and CRP; referred to as “metabolic-inflammatory markers” below). To provide insights into regional neural substrates of brain age, we then employed an explainable artificial intelligence (XAI) method to identify areas contributing significantly to the age predictions of the convolutional neural network model used for this purpose in our study. In the first main analysis, we tested links between the four metabolic-inflammatory markers and brain-PAD. In the second, we tested links between brain-PAD and performance in neuropsychological tests for the MMS to evaluate the clinical utility of weight loss-induced brain-PAD variations.

## Methods

### Participants

**Fig. 1:**
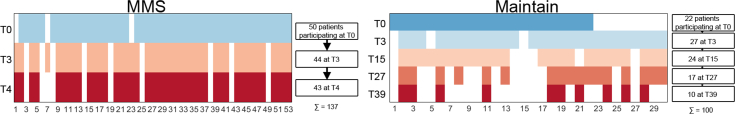
**Data availability by trial across time.** The figure illustrates the availability of MRI data per timepoint and dataset. Coloured tiles indicate the participation of a person, white tiles indicate absence. MRI data were chosen as reference as the number of available data points for all other data types was maximally as large as that for MRI.

The “Muscle Metabolism Study” (MMS) was performed by the Clinic of Endocrinology, Diabetes and Metabolism at Charité—Universitätsmedizin Berlin, Germany from 2012 to 2017. The protocol included three time points: a baseline visit (‘T0’) comprising 50 participants with a complete MRI scan after application of inclusion and exclusion criteria (see Supplement) and follow-up visits after three (‘T3’, N = 44) and four (‘T4’, N = 43) months. Thus, overall, 137 samples were available for this trial that were taken from a total of 53 participants. Please see Fig. 1 for an illustration of the availability of data per person and timepoint. Participants were randomised into a weight intervention versus ad libitum group at the beginning of the trial. Participants in the intervention group participated in a weight loss diet from T0 to T3 and in a weight maintenance programme from T3 to T4. The study was approved by the Ethics Committee of the Charité University Medicine Berlin (EA2/050/10). All participants provided written informed consent. All study-related procedures were conducted in compliance with the International Conference on Harmonisation Guidelines for Good Clinical Practice and the Declaration of Helsinki. A detailed description of the study design was already published.^29^^,^^30^^,^^33^^,^^34^

The “Maintain” study was performed by the Clinic of Endocrinology, Diabetes and Metabolism at Charité—Universitätsmedizin Berlin, Germany from 2010 to 2016. The acquisition of imaging data was conducted by the Berlin Center for Advanced Neuroimaging (BCAN). The protocol included five time points: a baseline visit (‘T0’) comprising 22 participants with a complete MRI scan after application of inclusion and exclusion criteria (see Supplement) and follow-up visits after three (‘T3’, N = 27), 15 (‘T15’, N = 24), 27 (‘T27’, N = 17) and 39 (‘T39’, N = 10) months. Overall, 100 samples were available for this trial that were taken from a total of 30 participants (Fig. 1). From T0 to T3, all persons participated in a weight loss diet. Afterwards, they were randomised into a weight maintenance programme (lasting from T3 to T15) or ad libitum group at T3 (see section body weight modification). Metabolic-inflammatory markers were available for T0, T3 and T15. All persons provided written informed consent prior to participation. The study was approved by the Ethics Committee of the Charité—Universitätsmedizin Berlin (EA2/017/09; EA1/063/09) and conducted in accordance with relevant guidelines. The participants could receive up to 460€ for study participation. Data acquired in Maintain and analysed for different purposes were already analysed.^31^^,^^35^, ^36^, ^37^, ^38^

### Body weight modification

In the MMS, the weight-loss intervention consisted of a weekly counselling by clinical dieticians and using a structured weight reduction programme. In the first eight weeks participants underwent a total meal replacement programme using a low-caloric formula diet (800 kcal/day–Optifast 2®, Nestlé HealthCare Nutrition GmbH, Frankfurt/Main, Germany). After eight weeks the formula diet was switched to an energy-reduced diet with a balanced macronutrient composition in accordance with the guidelines of the German Nutrition Society (see http://www.dge.de). Participants were advised to increase physical activity to reach a goal of 150 min of activity per week. Subsequently, subjects were instructed to maintain their reduced weight for four weeks by adhering to an isocaloric diet. Participants in the ad libitum group were measured in parallel but instructed to keep their dietary habits constant during the study.

Similarly, in Maintain, the weight loss diet was divided into two phases: a very low-calorie formula diet (Optifast 2®; 800 kcal/day) lasting eight weeks conducted under medical supervision and a four-week period of a calorie-restricted standard diet (1500 kcal/d) composed according to the German Nutrition Society guidelines. Besides, the weight-loss diet comprised dietary supervision and counselling by a nutritionist and 30-min group meetings of the participants including cooking classes and guided physical exercises (such as aqua fitness or therapeutic exercise training). In the maintenance programme, participants met in 90-min group meetings from T3 to T15 on a regular basis comprising mild physical activity, nutritional counselling, and psychological relaxation techniques administered by a clinical psychologist. Persons included in the ad libitum group were only provided with a short advice leaflet.^35^

### Assessment of metabolic-inflammatory markers

Fasting blood samples (which were available for both trials for the first three visits) were centrifuged; plasma and serum samples were frozen immediately at −80 °C. Serum insulin was measured by fluoroimmunometric assay (AutoDelfia Insulin Kit; PerkinElmer, Rodgau, Germany; Cat# B080-101, discontinued) (inter-assay CV 2.3–3.5%, intra-assay CV 1.7–2.4%). Glucose was measured using the glucose oxidase method on a Super GL glucose analyser (Dr. Müller Super GL, Freital, Germany). The insulin resistance index HOMA index was calculated as previously described: fasting glucose (mg/dl) × fasting insulin (mU/L)/405.^39^ Leptin was assessed in serum samples using commercial ELISA (R&D Systems, Abingdon, UK, Cat# RD191001100, RRID:AB_3696585) (intra-assay CV: 3.0–3.3%, inter-assay CV: 3.5–5.4%). Fetuin-B was measured in serum samples by ELISA using a commercial ELISA kit (Human Fetuin B DuoSet ELISA; R&D Systems, Minneapolis, USA, Cat# DY1725, RRID:AB_3083709) (intra-assay CV: 5.3%, inter-assay CV: 8.2%). CRP was analysed by standard laboratory methods using Cobas c111 Analyser (Roche Diagnostics, Mannheim, Germany).

### Assessment of neuropsychological markers

In the MMS, participants’ capacities in the domains of memory, psychomotor speed, attention, and executive functioning were measured with neuropsychological instruments at T0, T3 and T4. Specifically, the German version of the Auditory Verbal Learning Test (VLMT)^40^ was applied to test verbal recognition memory. Here, participants were presented with a spoken list of 15 words in five consecutive trials each and were asked to learn as many words as possible. Immediately after each trial and after a 30-min delay the participants had to recall the words. Lastly, participants were required to select the previously learnt words from a list that also contained 15 terms from an interference list that they had learnt during the delay, as well as 20 new words. The recognition score of the VMLT corresponds to the number of correctly recognised words minus words that were recognised but not actually included in the list. In addition to this primary outcome measure of the VLMT, we evaluated three additional memory scores from this instrument, i.e., the “learning” (number of correctly recalled words across the immediate learning trials), “delayed recall” (number of words correctly recalled after the 30-min delay), and “consolidation score” (number of words correctly recalled after the fifth learning trial minus the number of correctly recalled words after the delay). In the test of digit span,^41^ which provides a measure of simple attention, the participants were presented with a sequence of digits and had to repeat them in the original order. The number of repeated digits was noted as test measure. In the Trail Making Test (TMT),^42^ which assesses psychomotor speed, visual attention, mental flexibility, and working memory, participants had to connect a series of numbers (Part A) and then connect alternating numbers and letters in ascending order (Part B) as fast as possible. For both parts, time to complete in seconds was noted as test measure. In the Stroop colour-word test,^43^ which provides a measure of executive functioning, the participants were required to name perceptual characteristics of stimuli presented on a list across three conditions. Here, we used the duration the participants needed in the third condition as test score in which they had to name the colour of the ink colour-words were written in that name another colours than that of the ink. Finally, in the phonetic and semantic verbal fluency test (Regensburger Wortflüssigkeitstest),^44^ which primarily measures executive functions and semantic memory participants had to name words beginning with the letters S and P for 1 min each to assess phonetic fluency, and first names and animals for 1 min each for semantic fluency. Test scores for phonetic and semantic fluency were then calculated using the mean value of the words named in both subtests each. All tests were administered by a trained clinical neuropsychologist blinded for group membership. Please note, that these neuropsychological data have also been analysed and reported in a separate publication by Prehn et al. (2016).^29^

### MRI acquisition

All MRI brain scans were acquired with a 3-D-magnetisation prepared rapid gradient echo (MPRAGE) MRI-sequence. Specifically, all images acquired in the MMS and all scans acquired in Maintain at time points T3 and later were measured with a 3 T whole-body tomograph (Magnetom Trio, Siemens, Erlangen, Germany) using a standard 12-channel head coil. The sequence employed had 192 slices and covered the whole brain (slice thickness 1 mm; in-plane voxel resolution 1 · 1 mm^2^; TR = 1900 ms; TE = 2.52 ms; FA = 9°; FOV = 256 · 256 mm^2^; matrix size = 256 · 256). Scans acquired at T0 in Maintain were measured with a 1.5 T whole-body tomograph (Magnetom Sonata, Siemens, Erlangen, Germany) using a 12-channel head coil. This sequence also had 192 slices and covered the whole brain (slice thickness 1 mm; in-plane voxel resolution 0.5 · 0.5 mm^2^; TR = 1970 ms; TE = 3.68 ms; FA = 15°; FOV = 256 · 256 mm^2^; matrix size = 512 · 512).

### MRI pre-processing

Pre-processing of MRI brain scans used for brain age computation can be divided in three steps. In the first, basic pre-processing steps were performed mainly using SPM12 (Wellcome Trust Centre for Neuroimaging, Institute of Neurology, UCL, London UK; http://www.fil.ion.ucl.ac.uk/spm). In the second, we performed a quantitative brain image quality assessment, in the third we determined the ventricular and intracranial volumes. Steps two and three were conducted with Freesurfer.^45^

The basic pre-processing of MRI scans for brain age computation with the algorithm of Bashyam et al. (2020)^46^ followed these authors' pre-processing steps. Specifically, first, we spatially co-registered our participants’ T1-weighted scans from their native image space to the anatomical reference space defined by the Montreal Neurological Institute (MNI)^47^ using SPM12 (Wellcome Trust Centre for Neuroimaging, Institute of Neurology, UCL, London UK; http://www.fil.ion.ucl.ac.uk/spm). Following the procedure employed by Bashyam et al. (2020) (46), only a linear transformation was applied in this mapping procedure. Next, we used a U-Net-based convolutional neural network (CNN) for skull-stripping (https://github.com/MICLab-Unicamp/CONSNet).^48^ Spurious voxels located outside the binary brain mask provided by SPM12 were also removed. The resulting images were used for brain age computation.

In the quantitative brain image quality assessment, we used a method proposed by Rosen et al. (2018)^49^ to characterise the quality of the MPRAGE scans used to calculate brain age. In particular, Freesurfer's recon-all was used to reconstruct the surface of each brain hemisphere separately first, the MRIS_EULER_NUMBER algorithm was then used to compute the Euler number for each hemisphere based on the original, unfixed, surface reconstruction and the average across Euler numbers computed for both hemispheres was finally determined as quality parameter for a given scan. This parameter was entered as nuisance factor in all linear (mixed) model analyses of brain age or brain-PAD.

Finally, the Estimated Total Intracranial Volume computed with recon-all based on the MPRAGE of each participant's visit was used as measure of the intracranial volume, and the sum of the volumes of left and right lateral and lateral inferior ventricle as well of the third and fourth ventricle also computed with recon-all was determined as measure of ventricular volume. Ventricular volume was included as nuisance factors in all analyses testing treatment effects on brain-PAD, associations between brain-PAD and metabolic and cognitive parameters and regional neural substrates of brain age. Intracranial volume was computed as additional nuisance factor included in analyses of regional neural substrates of brain age.

### Computation of brain-age and brain-PAD

Brain age was calculated for each participant and time point separately based on the pre-processed T1-weighted MPRAGE scans using the publicly available CNN (https://github.com/vishnubashyam/DeepBrainNet) of Bashyam et al. (2020),^46^ trained on a multi-centre dataset comprising 11,729 T1-weighted scans acquired in different MRI settings (e.g., using 1.5 and 3 T scanners). With a correlation of r = 0.98 between the predicted brain age and the chronological age, the algorithm reached an excellent prediction accuracy in a model validation procedure in their study. Specifically, the algorithm predicts brain age separately for individual axial brain slices and then determines the final brain age prediction as the median age prediction across slices. Brain-PAD was calculated by subtracting the chronological age from the predicted brain age.

### Computation of CNN-derived brain maps of voxel importance for age prediction

Here, we used an XAI method to compute brain maps for each person and visit coding the importance of each voxel for age prediction through the CNN model employed in our study^46^ to test regional contributions to prediction in a later group analysis (‘Regional neuronal substrates of brain age’; see below). The XAI approach employed, the so-called Shapley value method, is inferred from a game theoretical approach which aims to distribute the value of a “game” (here resembling the prediction of a machine learning model) among its “players” (here resembling the features in a model) in a fair fashion.^50^ Coarsely speaking, the Shapley value of a feature reflects the average impact of varying this feature on the model's prediction for a sample when all other features are held constant. Thus, it reflects the feature's impact on the deviation between the model's prediction for that specific sample and its average prediction across all samples.^51^ Positive Shapley values indicate features/voxels biasing the prediction of the model towards an older age prediction, negative values those biasing the model's prediction towards a younger age.

Specifically, to determine these Shapley value voxel importance maps, we applied the Shapley Additive exPlanations toolbox for Python by Lundberg & Lee (https://github.com/slundberg/shap)^52^ to the pre-processed T1-weighted scans used for brain age prediction (which were co-registered to the MNI-template using a linear co-registration procedure only; see MRI pre-processing). Afterwards, we used the deformations toolbox of SPM12 for two image co-registration steps. In the first, we inversely normalised the Shapley value maps from MNI space back to native image space based on the affine co-registration matrix determined during the linear-only normalisation step. In the second, we co-registered the resulting map to MNI template space using the deformation field generated by the Computational Anatomy Toolbox (CAT12)^53^ during a combined non-linear spatial normalisation and segmentation procedure applied to the raw T1-weighted scan of a participant and visit. The reason for applying this procedure was that nonlinear co-registration techniques achieve better overlap between the individual and the template image than linear-only procedures and are thus better suited for voxel-wise group analyses.^54^ After the resulting Shapley value maps were constrained to voxels located in a group brain mask of intracranial tissue generated for each trial separately, they were entered into the group analysis. For each trial, the group mask was generated by selecting only those voxels for which all Shapley-value maps of all participants and visits in the trial had non-zero values and were located in SPM12's brain mask at the same time. See also our recent brain age paper for an application of this Shapley-value based method in a brain age framework.^4^

### Statistical analysis

In the following, we describe analyses conducted to evaluate brain age prediction accuracy, treatment effects, investigate regional neural substrates of brain age, and to test associations between BMI, metabolic-inflammatory parameters, brain-PAD and parameters of cognitive functioning. All analyses conducted are longitudinal analyses in which the dependent variable (DV) and the predictor variables include the data of all available participants and visits. Except for analyses evaluating brain age prediction accuracy, for which a specific set of benchmark parameters was established which are not calculated with linear mixed models (LMMs), all analyses were conducted with LMMs. In all LMMs all fixed effect regressors except for the intercept were centred (i.e., had zero mean).

All analyses were conducted in R and MATLAB using established neuroimaging toolboxes (FreeSurfer, SPM12/CAT12, Shapley Toolbox; RRIDs as reported in Supplementary Information).

#### Accuracy of brain age prediction

To characterise the CNN's brain age prediction accuracy, we computed established parameters for this task, i.e., the root mean squared error between the chronological and predicted brain age, as well as the mean absolute error. As reference, we compute the standard deviation of chronological age.

#### Effects of participating in a weight management programme on BMI and brain-PAD

Here, we analysed BMI and brain-PAD trajectories with LMMs. BMI analyses had, however, only illustrative character as a weight reduction in both trials has been published before^30^^,^^32^ and was mandatory for inclusion in the randomised maintenance phase in Maintain. The LMMs for modelling BMI and brain-PAD were very similar. In particular, the LMMs for modelling BMI included fixed main effect regressors for group (intervention = 1, ad libitum = 0), chronological age at baseline, the presence of a metabolic syndrome (y/n), the application of diabetes drugs (y/n), a regressor coding the time after diet onset in days, another one the time after diet offset in days (and zeros for earlier time points) for both trials. For example, if the baseline/T0 visit took place 5 days prior to diet onset and the T3 visit took place 87 days after diet onset, the “time after diet onset regressor” would code −5 for T0 and 87 for the T3 visit for this person. Further, if the T3 visit took place 3 days after diet offset and the T15 visit 368 days after diet offset, this regressor would code 0 for the T0 visit, 3 for the T3 visit and 368 for the T15 visit in this person. The two time regressors were included to capture potentially non-linear effects of varying diet regimes during different temporal segments of the experimental design (e.g., a weight-loss diet from T0 to T3 and no intervention or weight maintenance from T3 to T15 in Maintain). This segmented regression method is a standard approach for modelling interrupted time series frequently observed in treatment studies.^24^^,^^55^ Further, statistically modelling well-known age effects on brain-PAD is a standard method for coping with such effects which can either be done by including one predictor for chronological age in the model, or, as in the present case, one regressor for age at baseline and another for time since diet onset.^56^ Additionally, all LMMs included the interaction regressor group × time after diet offset and a fixed intercept (capturing the mean across all samples) and a random intercept for subject (capturing the subject-specific means across visits). The LMMs for MMS and Maintain differed in that only that for MMS additionally included the interaction group × time after diet onset and only those for Maintain included a fixed main effect regressor for sex. For MMS, the two interaction regressors group × time after diet onset and group × time after diet offset served as covariates of interest (CI; because both groups differed in the diet regime in both temporal segments). For Maintain, the main effect regressor for time after diet onset and the interaction group × time after diet offset regressor served as CI (because both groups participated in a weight-loss diet). Modelling effects on brain-PAD was done almost identically, with the only difference being that the trial-specific LMMs additionally included fixed effects regressors for ventricular volume and image quality.

For each CI, we report the t-statistic and p-value. Specifically, for effects of initial weight loss from T0 to T3 (MMS: group × time after diet onset; Maintain: time after diet onset) we tested negative effects and thus performed one-sided tests. For later effects (MMS & Maintain: group × time after diet offset) we conducted two-sided tests as maintenance effects after weight loss and their direction are less clear than those for initial weight loss.^57^ Moreover, we report partial η^2^ (η^2^_p_) computed with the T_TO_ETA2 function of the EFFECTSIZE library for R^58^ as effect size measure for a given CI (η^2^_p_ ≥ 0.01 indicates a small, η^2^_p_ ≥ 0.06 medium, and η^2^_p_ = 0.14 a large effect).^59^ In these and all other LMM regression analyses conducted in this work, we excluded samples showing an outlier value for the tested CI (i.e., a value smaller than the first quartile minus 1.5 times the interquartile range or larger than the third quartile plus 1.5 times the interquartile range) to increase robustness. Therefore, and because not all measures serving as CI were available for all MRI samples, the evaluated number of samples could vary across analyses and is thus provided as additional parameter for each analysis.

#### Associations of BMI and brain-PAD

We determined the association between both parameters across time with LMM regression. The participants’ BMI served as fixed CI, fixed effect regressors for group, the presence of a metabolic syndrome, the application of diabetes drugs, chronological age, age at baseline, sex (Maintain only), ventricular volume and MR image quality, and a fixed and random intercept as covariates of no interest (CNI), and brain-PAD as DV. The significance threshold in one-sided test was α = 0.05.

#### Associations of BMI and metabolic-inflammatory markers

Associations between BMI and metabolic-inflammatory markers were analysed with the same LMM (in four analyses, one per marker) that was used for testing associations of BMI and brain-PAD, except that the MR-specific variables (ventricular volume and MR image quality) were not included here. To account for multiple comparisons, we used the False Discovery Rate (FDR) procedure of Benjamini & Hochberg^60^ per trial and defined the significance threshold of one-sided tests of positive effects to be α_FDR_ = 0.05. To determine multiple comparison adjusted confidence intervals for the fixed effect coefficients in the FDR framework, we used the false coverage-statement rate-method (FCR) proposed by Benjamini & Yekutieli.^61^ Noteworthy, FCR-adjusted confidence intervals for regression coefficients are only defined for significant effects.

### Regional neuronal substrates of brain age

In this analysis, we investigated which regions in the brain are most sensitive to ageing effects or respectively contribute significantly to the age predictions of the CNN model for brain age by testing whether the average Shapley values across participants and visits in a trial were significantly larger or smaller than zero per voxel. This was done by analysing the same LMM as used for testing effects of participating in a weight management programme on brain-PAD described above (plus a fixed effect regressor for the total intracranial volume) separately for each voxel based on all MRI data available for a trial. Given that all fixed effect regressors except for the intercept were centred, the coefficient obtained by the LMM for the intercept reflects the average Shapley-value per voxel and the corresponding t-statistic and p-value for this intercept indicate whether the average Shapley value is significantly larger or smaller than zero. The voxel-wise analyses were constrained to coordinates included in the trial-specific group brain masks described in the pre-processing section. We tested for negative effects (i.e., regions consistently biasing the model's prediction towards a younger age across participants and visits) and positive ones (biasing towards an older prediction). To ensure a maximally conservative adjustment for multiple comparisons in this voxel-wise analysis, we used the Bonferroni method and applied a family-wise error (FWE) corrected significance threshold. For both directions, the threshold was 0.025 (i.e., α_FWE-positive_ = α_FWE-negative_ = 0.025). To reduce spurious findings (relatively prominent in this analysis as we did not smooth the Shapley value maps to maintain a high spatial specificity), we report only those associations for which an association was found for at least 25 contiguous voxels.

#### Main analysis 1: associations of metabolic-inflammatory markers and brain-PAD

Associations between metabolic-inflammatory markers and brain-PAD were analysed with the same LMM (in four analyses, one per marker) that was used for testing associations of BMI and brain-PAD except that BMI at baseline was included as additional fixed CNI here. We again tested for positive associations in one sided tests and used the FDR method to adjust for multiple testing. Please note that, given that metabolic-inflammatory markers and MRI scans were not necessarily acquired on the same day due to the complex, multiparametric study design, Supplementary Analysis 1 tested associations between metabolic-inflammatory markers and brain-PAD controlled for this gap (Supplementary Figure S1).

#### Main analysis 2: associations of brain-PAD and neuropsychological test scores

To evaluate the clinical relevance of body weight modification-induced variations of brain-PAD, we tested associations between brain-PAD and the neuropsychological measures described above in participants of the MMS trial across visits. The LMMs computed for this purpose for each of the measures modelled brain-PAD as CI, the score in the given neuropsychological test as DV, and chronological age, age at baseline, ventricular volume and the MR image quality parameter plus fixed and random intercept as CNI. We hypothesised negative links between brain-PAD on one hand and digit span, both verbal fluency measures, VMLT: recognition, VMLT: learning, and VMLT: delayed recall on the other. For the remaining markers (i.e., both trail making tests, VLMT: consolidation, Stroop colour-word interference), we hypothesised positive associations. Consequently, we conducted one-sided tests. For instruments composed of several scales (i.e., VMLT, verbal fluency, TMT), the obtained p-values were adjusted for multiple comparison with the FDR method.

At this occasion, we want to highlight a set of additional analysis conducted in the Supplement. First, we conducted an analysis testing whether the results obtained on treatment effects and on associations between BMI, metabolic parameters, brain-PAD and neuropsychological parameters were affected by selection bias (i.e., drop out systematically depending on clinical variables; Supplementary Table S2). Moreover, we also conducted an analysis that repeated the analyses on treatment effects and on associations between BMI, metabolic parameters, brain-PAD and neuropsychological parameters based on only the data acquired for T0 and T3 to evaluate whether the deviations that existed for some associations between both trials are driven by differences in trial duration or other sources (Supplementary Table S3). Further, we conducted an analysis testing visit-wise association between metabolic-inflammatory markers and brain-PAD and between brain-PAD and cognitive functioning separately for individual visits (i.e., cross-sectionally) with classical linear regression models. This facilitates a rough estimation of the relative contribution of cross-sectional signal covariation to the longitudinal associations focused on in this work (Supplementary Figure S2). Finally, we also determined the statistical power of analyses testing treatment effects as well as associations between BMI, metabolic-inflammatory markers, brain-PAD and neuropsychological parameters (Supplementary Table S4).

### Role of funders

Funding sources were not involved in the design of the study; the collection, analysis, or interpretation of data; or the writing of the manuscript.

## Results

### Clinical and demographic participant characteristics

Please see Table 1 for detailed descriptive clinical and demographic participant characteristics.

**Table 1:** Clinical and demographic participant characteristics.

Trial/Parameter	Time point		
MMS	T0	T3	T4
Frequency measures			
N (→ MRI)	50	44	43
Intervention	24	23	23
Metabolic syndrome	41	36	34
Antidiabetic medication	4	3	1
Mean (standard deviations)			
Age	60.7 (5.2)	62.1 (4.9)	62.0 (4.8)
BMI	34.9 (4.2)	32.3 (4.4)	32.1 (4.5)
%Fat	44.6 (3.7)	42.0 (4.5)	42.3 (4.8)
Digit span	7.6 (1.7)	7.8 (2.0)	8.0 (2.2)
Verb. fluency, phon.	13.0 (3.1)	14.7 (3.2)	15.8 (3.9)
Verb. fluency, sem.	24.5 (4.4)	26.1 (5.5)	26.7 (5.2)
TMT part A	34.7 (10.9)	32.3 (11.9)	28.3 (9.0)
TMT part B	79.3 (30.7)	75.9 (25.6)	65.6 (22.9)
VLMT: recognition	11.6 (3.7)	12.5 (3.7)	12.1 (3.5)
VLMT: learning	50.3 (8.8)	57.4 (7.6)	57.6 (8.0)
VLMT: delayed rec.	9.9 (3.0)	11.0 (3.0)	11.3 (2.9)
VLMT: consol.	2.6 (1.5)	2.5 (2.1)	2.1 (1.9)
Stroop interf.	85.4 (20.6)	80.0 (19.7)	75.1 (16.8)

Maintain	T0	T3	T15	T27	T39
Frequency measures					
N (→ MRI)	22	27	24	17	10
Females	17	19	17	10	7
Intervention	9	12	11	8	4
Metabolic syndrome	10	9	9	6	2
Antidiabetic mediaction	0	1	0	1	0
Mean (standard deviations)					
Age	48.4 (14.0)	46.5 (13.5)	48.1 (14.4)	47.6 (14.3)	40.5 (12.3)
BMI	34.4 (3.1)	32.0 (3.9)	32.3 (3.7)	33.1 (4.2)	33.7 (5.5)
%Fat	33.5 (6.4)	31.3 (7.3)	30.9 (6.6)	NA	NA

Abbreviations: BMI, body-mass index; Con., consolidation; phon., phonemic; rec., recall; sem., semantic; Stroop interf., Stroop colour–word interference; syn., syndrome; verb., verbal; TMT, Trail Making Test; VLMT, Verbal Learning and Memory Test.

Clinical, anthropometric, and cognitive characteristics of participants in the MMS and Maintain trials at each assessment time point. Values are mean (standard deviation) unless otherwise indicated. “Frequency measures” denote counts of participants fulfilling the respective criterion at each time point. N (→ MRI) indicates the number of participants with MRI data available. For verbal fluency and all four VLMT measures, values correspond to the number of correctly produced or recalled words. For digit span, the number of correctly repeated digits is reported. Units for both TMT parts and Stroop interference are seconds. NA indicates that data were not available for the respective time point.

### Accuracy of brain age prediction

**Fig. 2:**
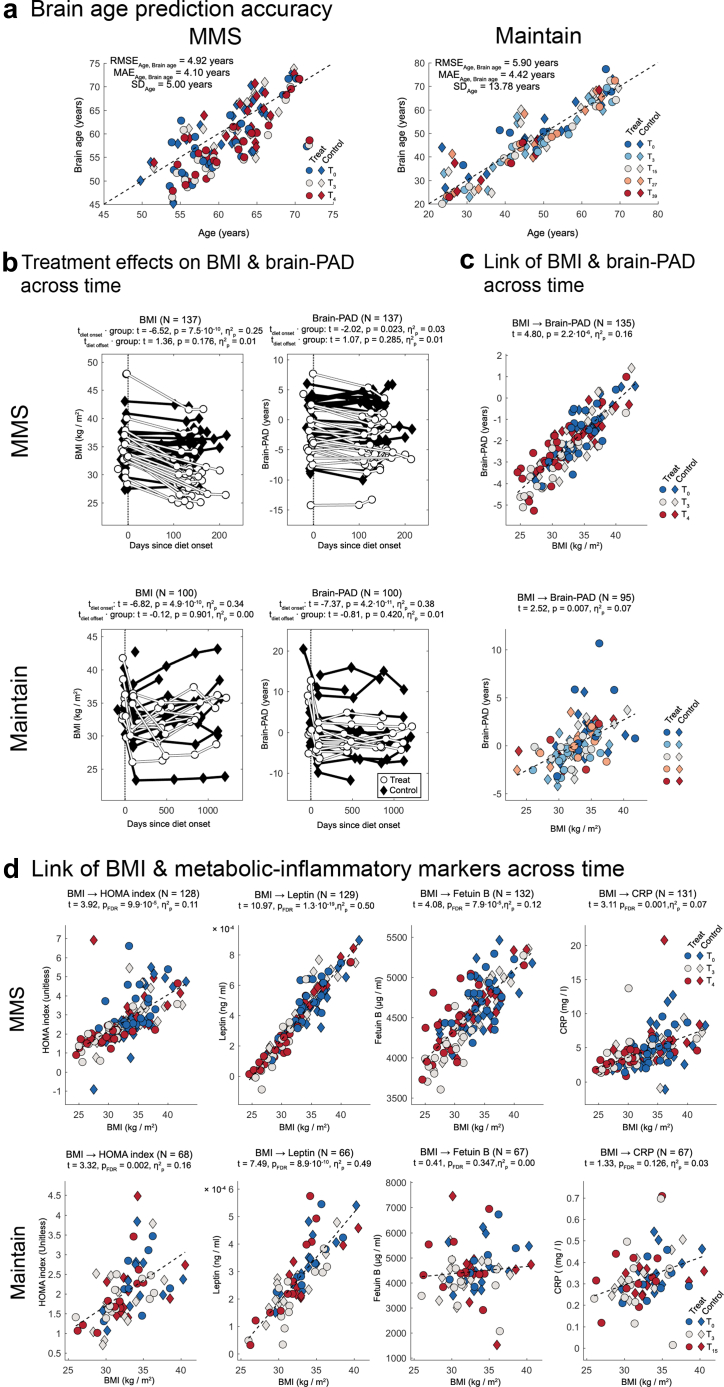
**Preparatory analyses.** (a) Scatterplots of predicted brain age versus chronological age for the MMS (left) and Maintain (right) cohorts. Root mean squared error (RMSE), mean absolute error (MAE) and standard deviation (SD) of chronological age are shown in each panel. (b) Individual trajectories of BMI (left) and brain-predicted age difference (brain-PAD; right) over time for MMS (top) and Maintain (bottom). Each line represents one participant. N corresponds to the total number of samples evaluated in the given analysis. Reported statistics summarise the effect of time since diet onset and the time × group interaction. (c) Association between BMI and brain-PAD across all available time points for MMS (top) and Maintain (bottom). In the scatter graphs depicted in this and all other figures, the values depicted for the DV on the ordinate axis are corrected for all CNI included in the given LMM. Consequently, only the mean but not necessarily the range of the DV are preserved across analyses due to potentially varying CNI across analyses. (d) Associations of BMI with metabolic-inflammatory markers across time. Top row: MMS; bottom row: Maintain. Scatterplots depict BMI versus HOMA index, leptin, fetuin-B and C-reactive protein (CRP), respectively. Colours encode measurement time point and symbols encode treatment versus control group. For all statistical annotations, *t* denotes the t-statistic, *p*_FDR_ the false discovery rate–corrected p-value, and η^2^p the partial eta-squared effect size.

For the MMS/Maintain trials the root mean squared error between predicted brain age and chronological age was 4.92/5.90 years, the mean absolute error 4.10/4.42 years. The standard deviation of chronological age computed as reference was 5.00/13.78 years in MMS/Maintain. Fig. 2a illustrates these results.

### Effects of participating in the weight management programme on BMI and brain-PAD

In MMS (in which only the treatment group participated in a weight loss diet), this diet induced a significant BMI reduction in the treatment relative to the ad libitum group from T0 to T3 (time since diet onset · group: t = −6.52, p = 7.5·10^−10^, β = −0.04, CI _95%_ = [−0.05 to −0.03], η^2^_p_ = 0.25). In Maintain (in which both groups participated in the weight loss treatment), both groups lost weight from T0 to T3 (time since diet onset: t = −6.82, p = 4.9·10^−10^, β = −0.04, CI _95%_ = [−0.05 to −0.03], η^2^_p_ = 0.34. Neither in the MMS (time since diet offset · group: t = 1.36, p = 0.176, β = 0.02, CI _95%_ = [−0.01–0.05], η^2^_p_ = 0.01) nor in the Maintain trial (time since diet offset · group: t = −0.12, p = 0.901, β = −1.3·10^−4^, CI _95%_ = [−2.2·10^−3^ to 2.0·10^−3^], η^2^_p_ = 0.00), participating in the weight maintenance group affected BMI significantly different than participating in the ad libitum control group.

Brain-PAD reductions were significantly larger in the treatment than the ad libitum group in MMS from T0 to T3 (time since diet onset · group: t = −2.02, p = 0.023, β = −0.02, CI _95%_ = [−0.03 to −3.6·10^−4^], η^2^_p_ = 0.03). Compatible with the fact that both groups participated in a weight loss programme in Maintain from T0 to T3, participants reduced brain-PAD in this period independent of group membership in Maintain (time since diet onset: t = −7.37, p = 4.2·10^−11^, β = −0.06, CI _95%_ = [−0.08 to −0.05], η^2^_p_ = 0.38). Neither in the MMS (time since diet offset · group: t = 1.07, p = 0.285 β = 0.02, CI _95%_ = [−0.02–0.05], η^2^_p_ = 0.01) nor in the Maintain trial (time since diet offset · group: t = −0.81, p = 0.420 β = −9.0·10^−4^, CI _95%_ = [−3.1·10^−3^ to 1.3·10^−3^], η^2^_p_ = 0.01), participating in the weight maintenance group affected brain-PAD significantly different than in the ad libitum control group (Fig. 2b).

### Associations of BMI and brain-PAD

BMI showed a significant positive link with brain-PAD across the full duration of both trials (MMS: four months, Maintain: 39 months; Fig. 2b). Specifically, for MMS, we determined t = 4.80, p = 2.2·10^−6^, β = 0.28, CI _95%_ = [0.16–0.39], η^2^_p_ = 0.16 and for Maintain t = 2.52, p = 0.007, β = 0.35, CI _95%_ = [0.07–0.62], η^2^_p_ = 0.07 (Fig. 2c).

### Associations of BMI and metabolic-inflammatory markers

BMI showed a strong and significant positive association to all metabolic-inflammatory markers except for fetuin B and CRP in Maintain. Specifically, for the link of BMI and HOMA index, we calculated for MMS: t = 3.92, p_FDR_ = 9.9·10^−5^ β = 0.19, CI_FCR95%_ = [0.11–0.27], η^2^_p_ = 0.11 and for Maintain: t = 3.32, p_FDR_ = 0.002, β = 0.13, CI_FCR95%_ = [0.05–0.20], η^2^_p_ = 0.16). Furthermore, for leptin, we computed for MMS: t = 10.97, p_FDR_ = 1.3·10^−19^, β = 4.8·10^3^, CI_FCR95%_ = [4.0·10^3^–5.7·10^3^], η^2^_p_ = 0.50 and for Maintain: t = 7.49, p_FDR_ = 8.9·10^−10^, β = 3.2·10^3^, CI_FCR95%_ = [2.4·10^3^–4.1·10^3^], η^2^_p_ = 0.49). In MMS, we determined for the link between BMI and fetuin B in MMS: t = 4.08, p_FDR_ = 7.9·10^−5^, β = 73.8, CI_FCR95%_ = [43.9–103.9], η^2^_p_ = 0.12 and in Maintain: t = 0.41, p_FDR_ = 0.347, β = 26.1, CI_FCR95%_ = [n.d.], η^2^_p_ = 0.00). Finally, for CRP we calculated in MMS: t = 3.11, p_FDR_ = 0.001, β = 0.32, CI_FCR95_ = [0.15–0.49], η^2^_p_ = 0.07 and in Maintain: t = 1.33, p_FDR_ = 0.126, β = 0.01, CI_FCR95%_ = [n.d.], η^2^_p_ = 0.03). Fig. 2d provides further details.

### Regional neuronal substrates of brain age

**Fig. 3:**
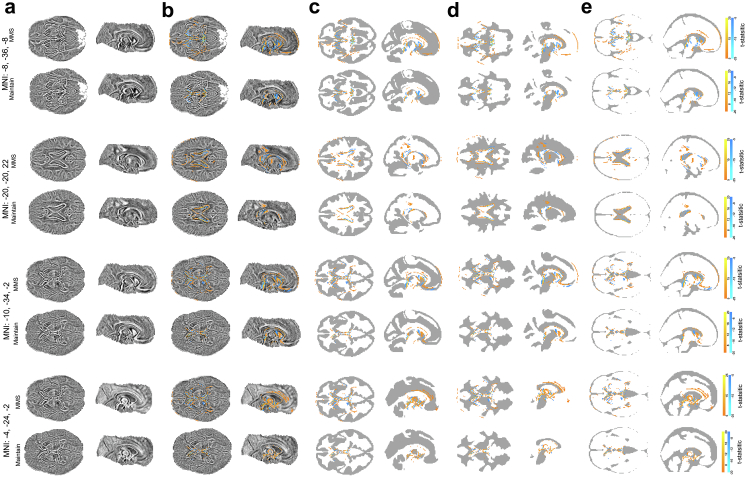
**Regional neuronal substrates of brain age.** (a) Panel depicts the results of our voxel-wise LMM analysis of Shapley-value brain maps in terms of the t-statistics obtained by the LMMs for the fixed intercept. Specifically, in groups of two rows, a) depicts (from top to bottom) the axial and sagittal brain slices centred on the coordinate with the strongest positive t-statistic in MMS (MNI: −8, −36, −8; i.e., brain stem) separately for MMS and Maintain, the strongest positive t-statistic in Maintain (MNI: −20, −20, 22; caudate nucleus), as well as the strongest negative t-statistic in MMS (MNI: −10, −34, −2; thalamus proper) and Maintain (MNI: −4, −24, −2; ventral diencephalon). From dark to bright, the voxel intensities indicate negative to positive t-statistics. As we intend to provide a pure insight into the distribution of ageing-related brain regions, we did not additionally highlight significance in this panel. (b) Panel shows voxels with significant positive/negative t-statistic in orange/blue (α_FWE-positive_ = α_FWE-negative_ = 0.025; 25 contiguous voxels) (c–e) Panels show the significant coordinates superimposed on binary masks for these tissues to allow associating the significant coordinates to GM (c), WM (d), and CSF (e). These masks were generated based on the tissue template included in SPM12 and defined in MNI-space by assigning a voxel to that tissue class for which it had the maximal probability. The orange/blue scale of t-statistics was identical across panels (b–e). Note: The model of Bashyam et al. (2020)^46^ covers only 80 axial slices in the centre of the MNI reference brain for an isotropic voxel-resolution of 1 mm^3^ which leads to missing Shapley values in superior and inferior brain regions.

Distributed brain regions contributed to brain age predictions performed by the CNN. Specifically, besides widespread white matter regions, in particular deep brain nuclei, cingulate, parahippocampal, frontal and temporal regions contributed amongst grey matter regions. Finally, also coordinates in ventricular regions in MNI-space located in direct vicinity to GM and WM were identified by this analysis. See Fig. 3 and Supplementary Table S1 for details.

### Main analysis 1: associations of metabolic-inflammatory markers and brain-PAD

**Fig. 4:**
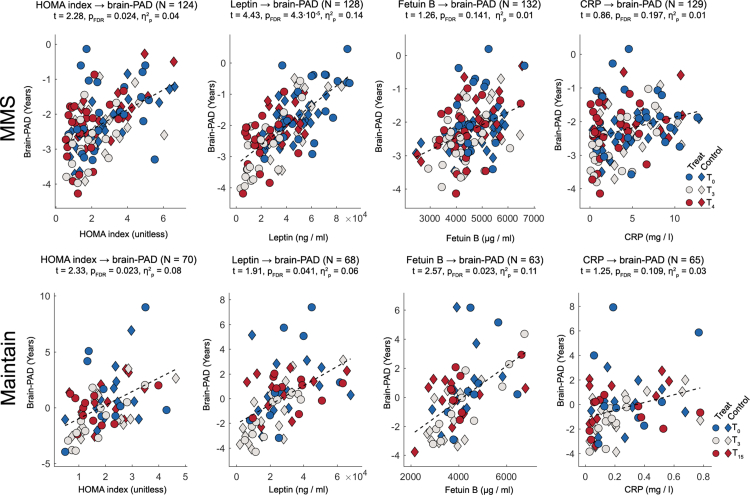
**Associations of metabolic-inflammatory markers and brain-PAD.** Scatter plots depict the relationship between brain-PAD (y-axis; years) and metabolic-inflammatory markers (from left to right) HOMA index, leptin, fetuin-B, and C-reactive protein (x-axes) for the MMS cohort (top row) and the Maintain cohort (bottom row). The metabolic-inflammatory markers (covariates of interest) depicted on the x-axes are raw values. Brain-PAD (dependent variable, DV) depicted on the y-axes is corrected for all predictors in a given model except for the covariates of interest and the fixed intercept (retaining the fixed intercept ensures that the average value in the corrected DV is the same as that of the uncorrected DV). For all statistical annotations, *t* denotes the t-statistic, *p*_FDR_ the false discovery rate–corrected p-value, and η^2^p the partial eta-squared effect size.

This analysis showed a significant positive association between HOMA index as well as leptin with brain-PAD for MMS and a significant positive association between HOMA index, leptin and fetuin B on one hand and brain-PAD on the other in Maintain. Specifically, for the link between HOMA index and brain-PAD we calculated for MMS: t = 2.28, p_FDR_ = 0.024, β = 0.28, CI_FCR95%_ = [0.04–0.53], η^2^_p_ = 0.04 and for Maintain: t = 2.33, p_FDR_ = 0.023, β = 1.22, CI_FCR95%_ = [0.27–2.17], η^2^_p_ = 0.08. Further, for leptin, we calculated in MMS t = 4.43, p_FDR_ = 4.3·10^−5^, β = 3.2·10^−5^, CI_FCR95%_ = [1.7·10^−5^–4.6·10^−5^], η^2^_p_ = 0.14 and in Maintain: t = 1.91, p_FDR_ = 0.041 β = 7.7·10^−5^, CI_FCR95%_ = [3.9·10^−6^–1.5·10^−4^], η^2^_p_ = 0.06. In MMS, we determined for fetuin B t = 1.26, p_FDR_ = 0.141, β = 4·10^−4^, CI_FCR95%_ = [n.d.], η^2^_p_ = 0.01 and in Maintain t = 2.57, p_FDR_ = 0.023, β = 1.2·10^−3^, CI_FCR95%_ = [3.4·10^−4^–2.0·10^−4^], η^2^_p_ = 0.11. Finally, for CRP, we calculated in MMS t = 0.86, p_FDR_ = 0.197, β = 0.06, CI_FCR95%_ = [n.d.], η^2^_p_ = 0.01 and in Maintain: t = 1.25, p_FDR_ = 0.109, β = 3.17, CI_FCR95%_ = [n.d.], η^2^_p_ = 0.03 (Fig. 4).

### Main analysis 2: associations of brain-PAD and neuropsychological test scores

This analysis identified a positive association significant according to a multiple comparison-corrected significance level between brain-PAD and TMT, part A across time for participants in the MMS trial (t = 2.32, p_FDR_ = 0.022, β = 0.67, CI_FCR95%_ = [0.10–1.24], η^2^_p_ = 0.05). Due to the number of parameters per evaluated neuropsychological parameter, we would like to point the reader to Table 2 for the details on the other tests.

**Table 2:** Associations of brain-PAD and neuropsychological test scores.

Parameter	t	p	b	CI	η^2^_p_
Digitspan	−1.50	0.069	−0.08	−0.20 to 0.03	0.02
Verb. fluency, phon.	0.20	0.580	0.02	n.d.–n.d.	0.00
Verb. fluency, sem.	−1.31	0.193	−0.18	n.d.–n.d.	0.02
TMT part A	2.32	0.022	0.67	0.10–1.24	0.05
TMT part B	0.29	0.387	0.18	n.d.–n.d.	0.00
VLMT: recognition	−0.52	0.303	−0.05	n.d.–n.d.	0.00
VLMT: learning	−0.58	0.303	−0.14	n.d.–n.d.	0.00
VLMT: delayed rec.	−1.18	0.303	−0.10	n.d.–n.d.	0.01
VLMT: consol.	0.87	0.303	0.04	n.d.–n.d.	0.01
Stroop interf.	0.05	0.483	0.03	−1.04 to 1.09	0.00

Note: For instruments comprising more than one scale, p corresponds to the p-value corrected for multiple comparisons using the False Discovery Rate (FDR) method (α_FDR_ = 0.05), and CIs correspond to 95% confidence intervals adjusted using the False Coverage Rate (FCR) method (CI_FCR95%_; Benjamini & Yekutieli, 2005^61^). For instruments with a single scale, uncorrected p-values and standard 95% CIs are reported. Within the FCR method, CIs are only defined for parameters that remain significant after correction; therefore, confidence intervals are not defined (n.d.) for non-significant parameters.

Abbreviations: b, unstandardised regression coefficient; CI, confidence interval; CI _95%_, 95% confidence interval adjusted for False Coverage Rate; consol., consolidation; η^2^p, partial eta-squared; n.d., not defined; p, p-value; phon., phonemic; rec., recognition; sem., semantic; Stroop interf., Stroop colour–word interference; t, t-statistic; TMT, Trail Making Test; verb., verbal; VLMT, Verbal Learning and Memory Test.

Shown are t-statistics (t), p-values (p), unstandardised regression coefficients (b), confidence intervals (CI), and partial eta-squared (η^2^p) for the association between brain-PAD and each neuropsychological measure.

## Discussion

We investigated the effect of metabolic–inflammatory processes on brain health characterised by brain-PAD and cognitive functioning in persons with obesity participating in two independent dietary weight modification trials. The results in both trials revealed converging evidence for a beneficial impact of weight loss-triggered improvements in metabolic-inflammatory markers on brain-PAD which in turn were associated with better cognitive functions.

Our study comprised five preparatory and two main analyses. In the first preparatory analysis, we evaluated the accuracy of the CNN model^46^ in predicting brain age from T1-weighted MPRAGE scans. This showed, that the accuracy, which was calculated in an out-of-sample framework, was well comparable across trials and with that determined in related studies of other authors.^62^

The second analysis tested whether one prerequisite for our study was fulfilled, i.e., whether participation in the weight modification programme affected BMI and brain-PAD. Although evaluating effects on BMI had illustrative character, it again documented that weight modification was successful in both trials with respect to the selected cohorts (i.e. in participants in which a structural MRI was available at the respective timepoint). Furthermore, we found in both trials that a three-months weight loss diet led to significant improvements in brain health reflected by brain-PAD. At this point, we would like to mention that while a basic effect of weight reduction on brain-PAD is consistent with prior work,^27^^,^^28^ the fact that a reduction of brain-PAD already followed three months after diet onset is not consistent with Zeighami et al. as the earliest effect found in their work was twelve months after bariatric surgery.^28^ Levakov et al. do not report effects for this short treatment period.^27^ Presumably, this difference is best explained by the fact that Zeighami et al.^28^ exclusively used grey matter signals for brain age prediction whereas our model used age-related information from grey and white matter (see Fig. 3 and Supplementary Table S1). Thus, one might assume that white matter processes contributing to brain age prediction are affected earlier by weight loss and underly this difference.

The third tested effects of BMI-variations across periods of weight loss, maintenance (Maintain: and regain) on brain-PAD which showed strong associations across the whole trial period for both trials. Given that the measurement interval (MMS: four months, Maintain: 39 months) and sample compositions differed substantially across trials (MMS included postmenopausal women only), the results thus suggest that the link between both factors is relatively robust.

The fourth analysis tested whether variations in metabolic-inflammatory markers reflect BMI variations triggered by the body weight modification programmes. Consistent with highly established findings,^63^, ^64^, ^65^ BMI showed a significant positive association to all metabolic-inflammatory markers on a multiple comparison-corrected level in both trials (except for fetuin B and CRP in Maintain).

Finally, in the fifth preparatory analysis, we investigated which regions in the brain are most sensitive to ageing effects i.e. contribute most strongly to the age predictions of the CNN model for brain age. This was done by testing where in the brain parameters from the field of XAI, so-called Shapley values, which indicate the importance of a feature for a model, are significantly larger or smaller than zero. Strikingly, since the CNN brain age model was by no means specialised in detecting obesity effects or to infer brain age from such effects, the regions identified in our study overlapped strongly with areas found in work that explicitly did search for regional obesity effects. Specifically, in line with the large MRI study of Janowitz et al.^66^ (>2000 participants) on grey matter correlates of obesity, these areas consistently included deep brain nuclei (caudate nucleus and putamen), cingulate cortex, and parahippocampal gyrus across both trials. Also, in line with Janowitz et al.,^66^ thalamus, frontal areas (amongst others middle frontal gyrus, precentral gyrus, and orbitofrontal cortex), and temporal lobes were prominently found in the analysis of the MMS data, which had slightly better power. Thus, taken together, the fact that regions identified by studies explicitly searching for regional grey matter correlates of excessive body weight^66^^,^^67^ overlap substantially with those found in our study as neural substrates of generic brain health might suggest that heightened BMI is an important general contributor to poor brain health. Furthermore, the fact that transition areas between grey or white matter on one hand and ventricular/cerebrospinal fluid areas on the other were frequently considered relevant by the CNN for age predictions (see Fig. 3) suggests that grey and white matter thinning or atrophy respectively is a key driver of brain ageing from the perspective of the employed deep learning model.

In main analysis 1, we addressed the key question of the present work, i.e., whether body weight modification-induced variations in metabolic-inflammatory markers are related to brain-PAD. This revealed significant associations for HOMA index and leptin across both trials on an α_FDR_ = 0.05 significance threshold. Fetuin B achieved this threshold in Maintain. Because the acquisition of metabolic-inflammatory markers and MRI did not always take place at the same day, we then conducted Supplementary Analysis 1 which recalculated the associations for controlled acquisition delays. This showed that the pattern of associations largely remained unchanged when the delay was controlled (Supplementary Figure S1). Only for the shortest tested delay of 14 days in Maintain (for which only quite few samples were available) the associations became insignificant. Thus, in line with findings showing that insulin resistance and metabolic dysfunction is associated with reduced brain health in obesity and T2DM in observational studies,^68^^,^^69^ and given that the link between metabolic-inflammatory markers and brain-PAD spanned stages of weight loss and maintenance in our study, this suggests that body weight modification-induced metabolic-inflammatory improvements can contribute to improved brain health.

Amongst mechanisms potentially mediating this effect, low-grade inflammation might mediate the association between metabolic dysfunction and neurodegeneration.^70^ Impaired metabolic signalling involves cross-talk between different tissues characterise by hepatokine (fetuin B) and adipokine (leptin) secretion modulating insulin action.^6^ The effect of impaired insulin signalling on the brain might be mediated by alterations in insulin permeability of the blood–brain barrier, as well as increased in advanced glycation end products and amyloidogenesis, and decreases in vascular functioning.^71^^,^^72^ Leptin levels might contribute to such effects by regulating a chronic low-grade inflammatory state in obesity due to its function as a proinflammatory cytokine.^15^^,^^16^

Finally, main analysis 2 tested associations between brain-PAD and cognition using digit span, verbal fluency test, TMT, VLMT, and a Stroop colour word interference test. These analyses identified a significant positive link for the time needed for completion of the TMT (part A). A positive link between TMT (part A) and brain-PAD (i.e., the higher brain-PAD, the longer the patients need to connect numbers on a page with a pencil) fits well to a set of brain regions (i.e., caudate nucleus, middle frontal gyrus, precentral gyrus) not only found in the analysis of regional neural substrates of brain age in this study but also results of studies on functional neural substrates of TMT execution.^73^^,^^74^ Taken together, this analysis indicates that improvement in brain health linked to improvements in metabolic-inflammatory functioning have also beneficial effects on cognition.

In addition to these analyses presented in the main text (and Supplementary Analysis 1, which has already been mentioned above), several additional supplementary analyses were conducted. Specifically, in Supplementary Analysis 2 we tested putative effects of selection bias on the observed results by repeating the analysis presented in the main text based on data of only those patients who consistently participated in all visits of a trial evaluated in a given analysis. This revealed a strong overlap between results obtained for analyses conducted based on all available patient data and those conducted based on data of patients consistently participating in all visits exclusively. Only for two individual analyses in Maintain (i.e., associations between BMI and brain-PAD and between leptin and brain-PAD) the results were considerably weaker for the analyses based on patients participating in all visits (Supplementary Table S2). However, given that the directionality of effects maintained stable in these cases and that constraining the data to patients participating in all visits was accompanied by a marked reduction in statistical power in Maintain, these two isolated findings do not question that, overall, our results are independent of effects of selection bias.

Moreover, in Supplementary Analysis 3, we repeated the analyses on treatment effects and on associations between BMI, metabolic parameters, brain-PAD and neuropsychological parameters across only the data acquired for T0 and T3 to evaluate whether the deviations that existed for some associations between both trials were driven by differences in trial duration or other sources. This showed that treatment effects on BMI and brain-PAD were largely identical for the temporally constrained data (i.e., acquired at T0 & T3) in MMS and Maintain. However, other than for MMS, the strength of the association between BMI and brain-PAD reduced strongly after constraining analyses to data of the two initial visits for Maintain. Despite such deviations however, one aspect was quite robust across all investigated effects and associations: the relations between effects/associations of interest calculated for MMS and Maintain based on the temporally constrained data were quite comparable to the relations of these effects/associations when computed based on the data from the full trial periods (Supplementary Table S3). Consequently, when deviations in effects between both trials existed these were presumably not driven by differences in trial duration but rather by other differences such as patient composition.

Supplementary analysis 4 tested associations between metabolic-inflammatory markers and brain-PAD and between brain-PAD and neuropsychological test scores separately for individual visits/cross-sectionally with classical linear regression models (Supplementary Figure S2a–c). We choose this approach to test associations for each visit individually to provide a rough insight into the relative contribution of cross-sectional signal covariation to the longitudinal associations focused on in this work. Although the numerical directionality of associations was compatible with that of the corresponding LMM analyses in several cases, the results were not significant according to the FDR threshold applied. A complementary analysis (Supplementary Figure S2d), however, suggested that these limited signal detection properties rely on the inability of classical linear regression models to correct for random between-subject factors (such as the participants’ mean signal/the random intercept for subject in the present case). Thus, with the caveat that statistical power was also lower for the cross-sectional analyses, the fact that models exclusively relying on between-subject signal covariation (the linear models testing visit-wise associations) failed to identify significant associations but that longitudinal associations could be detected with the proper methods (employed by LMMs) argues that longitudinal processes and signal covariation across different periods of weight modification programmes are the key drivers of the associations found in main analysis 1.

Ultimately, Supplementary Analysis 5 tested the sensitivity of analyses evaluating treatment effects as well as associations between BMI, metabolic parameters, brain-PAD and neuropsychological parameters in post hoc power analyses. Overall, the observed power/power determined post hoc computed for rapid treatment effects on BMI and brain-PAD, for associations between BMI and brain-PAD, as well as for the associations between BMI and metabolic parameters was adequate to high. Among analyses testing associations between metabolic parameters and brain-PAD, however, only the observed power for leptin and brain-PAD in MMS was very good and for the associations between brain-PAD and neuropsychological test scores, none reached adequate levels (Supplementary Table S4). Thus, from this perspective, one might argue that our study was lacking statistical power to identify a larger set of associations between metabolic parameters and brain-PAD (although five out of eight tested associations were significant on a threshold corrected for multiple comparisons) and even more so between brain-PAD and neuropsychological tests. And this argument cannot be disputed with pre-study power calculations, because, although both trials were of course grounded on solid pre-study sample size calculations, these were not performed for the associations tested here.

However, at the same time, it is true that the overall utility of observed or post hoc power is currently under debate as it may not be a good estimator of true power (e.g., due to experimental context factors or unmeasured variables) as Heinsberg & Weeks^75^ could show in a simulation study. Specifically, in this study, the authors were able to show that two identical experiments with identical p-values (p = 0.052) but different true power (which can be easily determined in a simulation study) can be assessed as uniformly underpowered based on post hoc power, since the latter is inferred 1:1 from the observed p-value. For this reason, post hoc power cannot differentiate between studies having missing results due to limited true power vs. those having missing results due to the absence of true effects. Consequently, even large studies can appear underpowered when characterised based on observed power. Thus, inferred from these findings and arguments we would like to draw the cautious conclusion that the power of our study appears sufficient for detecting treatment effects, associations between BMI and brain-PAD, as well as associations between some metabolic factors and brain-PAD but especially for detecting associations between brain-PAD and neuropsychological tests, larger samples could have been preferable.

Several limitations of the study have to be considered. A first point is that (other than in the MMS trial) the MRI setting in Maintain varied across the course of the trial (i.e., from T0 to T3): Although the MRI scans used for brain age computation were acquired with a standard T1-weighted MPRAGE sequence throughout the course of this trial, the scanner at T0 was a 1.5 T S Sonata scanner and a 3.0 T S Trio scanner at all later time points. Consequently, brain-PAD evaluated in Maintain might be differently affected by MRI effects for the Maintain than the MMS trial. However, we assume that these effects are not critical for two reasons. First, as indicated by Fig. 2b, the association between BMI and brain-PAD was strong not only in the Maintain trial but also (and even more so) in the MMS trial and all scans in MMS were acquired within constant MRI settings. Second, the CNN of Bashyam et al.^46^ used for brain age computation was intentionally trained on T1-weighted MRI scans acquired in heterogeneous MRI settings to provide a brain age prediction platform highly robust towards variation in such settings. Second, the fact that the CNN model of Bashyam et al.^46^ does not cover the entire brain but (only) 80 slices located around the centre of the brain in MNI standard space did prevent computing voxel-wise Shapley values for coordinates located in superior (e.g., the most superior regions of frontal cortex) or inferior (such as the cerebellum) parts of the brain. Consequently, it was not possible to compute the contribution of these regions to brain age calculation performed by the CNN model either. Finally, our cohorts are not representative of the full spectrum of obesity, particularly morbid obesity (BMI ≥40 kg/m^2^), nor of the general population. However, the inclusion criteria of both trials closely mirror current clinical thresholds for initiating obesity treatment, indicating that our findings are well representative of patients typically entering clinical care and thus have direct relevance for clinical practice.

Taken together, our work suggests beneficial effects of body weight modification-triggered improvements in metabolic-inflammatory functioning on brain health of obese participants of short and long-term weight management programmes. Improvements in brain health linked to those in metabolic-inflammatory functioning and BMI have also beneficial effects on cognition.

## Contributors

Conceptualisation (MW, LM, AF, JH, JS, KM); Formal analysis (LM, LK, LH, MW); Funding acquisition (JS, KM, JH, AF); Methodology (MW, LM, KM); Project administration (JS, KM, JH, AF); Supervision (MW, JS, KM, JH, AF); Visualisation (MW, LM); Writing—original draft (MW, LM); Writing—review & editing (LM, LK, LH, AF, JH, JS, KM, MW). LM and MW accessed and verified the data; all authors had full access and accept responsibility for the decision to submit.

## Data sharing statement

The DeepBrainNet model used for brain age calculation is publicly available via Github (https://github.com/vishnubashyam/DeepBrainNet). Pseudonymized tabular data for clinical and laboratory variables as well as cognitive test results including raw outputs of the brain-age analysis and the MATLAB code used for the analysis of the tabular data is made publicly available via Zenodo (https://zenodo.org/records/17540342 https://doi.org/10.5281/zenodo.17540342).

## Declaration of interests

All authors declare no competing interests.
