# Supplementary material for: Metabolic inflammation, brain age and cognitive functioning in short- and long-term clinical weight loss trials
Source: eBioMedicine. 2025 Dec 2;123:106064. doi: 10.1016/j.ebiom.2025.106064 (PMC12719091; doi:10.1016/j.ebiom.2025.106064)
Supplement: Supplementary Information [file mmc1.docx]

**Metabolic inflammation, brain age and cognitive functioning in short- and long-term clinical weight loss trials**

Maurer Lukas^1,2, †^, Kozarzewski Leonard^1,2^, Haberbosch Linus^1,2,3^, Flöel Agnes^4^, Haynes John-Dylan^5,6,7,8,9^, Spranger Joachim^1^, Mai Knut^1,10^*, Weygandt Martin^5,6,7^*, ^†^

**SUPPLEMENTARY INFORMATION**

^1^ Charité – Universitätsmedizin Berlin, corporate member of Freie Universität Berlin, Humboldt-Universität zu Berlin, and Berlin Institute of Health; Department of Endocrinology and Metabolism; European Reference Network on Rare Endocrine Diseases (ENDO-ERN), 10117 Berlin, Germany.

^2^ Pituitary Tumor Center of Excellence at Charité – Universitätsmedizin Berlin, corporate member of Freie Universität Berlin, Humboldt-Universität zu Berlin, and Berlin Institute of Health, Charitéplatz 1, 10117 Berlin, Germany

^3^ Cambridge Endocrine Molecular Imaging Group, Institute of Metabolic Science, University of Cambridge, National Institute for Health Research Cambridge Biomedical Research Centre, Addenbrooke's Hospital, Hills Road, Cambridge, CB2 0QQ, UK

^4^ Department of Neurology, University Medicine Greifswald, Greifswald 17475, Germany

^5^ Experimental and Clinical Research Center, a Cooperation between the Max Delbrück Center for Molecular Medicine in the Helmholtz Association and Charité Universitätsmedizin Berlin, Berlin, Germany

^6^ Charité—Universitätsmedizin Berlin, Corporate Member of Freie Universität Berlin and Humboldt-Universität zu Berlin, Experimental and Clinical Research Center, Lindenberger Weg 80, 13125 Berlin, Germany

^7^ Max Delbrück Center for Molecular Medicine in the Helmholtz Association (MDC), Berlin, Germany

^8^ Cluster of Excellence NeuroCure, Charité – Universitätsmedizin Berlin, corporate member of Freie Universität Berlin, Humboldt-Universität zu Berlin, and Berlin Institute of Health, NeuroCure Clinical Research Center, 10117 Berlin, Germany

^9^ Bernstein Center for Computational Neuroscience and Berlin Center for Advanced Neuroimaging and Clinic for Neurology, Charité Universitätsmedizin, corporate member of Freie Universität Berlin, Humboldt Universität zu Berlin, and Berlin Institute of Health, Berlin, Philippstraße 13, Haus 6, 10115, Germany

^10^ Department of Human Nutrition, German Institute of Human Nutrition Potsdam-Rehbrücke, Nuthetal, Germany

* These authors contributed equally to this work.

† Corresponding authors: Lukas Maurer, Email: [lukas.maurer@charite.de](mailto:lukas.maurer@charite.de), Martin Weygandt, Email: [martin.weygandt@mdc-berlin.de](mailto:martin.weygandt@mdc-berlin.de)

**Methods**

**Inclusion and exclusion criteria**

In the MMS, we included postmenopausal women with a body mass index (BMI) > 27 kg/m². Exclusion criteria were history of severe untreated medical, neurological, and psychiatric diseases (i.e. unstable coronary heart disease, kidney and liver disease, systemic infections, endocrinological disorders, and severe arterial hypertension) as well as MRI-related contraindications and a significant change in dieting or smoking habits during the last three months. A total of 80 overweight or obese female subjects were initially included in the complete study, 53 in the MRI sub study.

In order to be included in Maintain, participants had to be at an age between 18 and 70 years and a BMI of at least 27. Individuals who had changed their weight by more than 5 kg in the two months prior to study enrolment or who had taken part in another diet study during this time were not allowed to participate. Only those participants who lost at least 8% from T0 to T3 were eligible to continue the programme after the diet. If so, participants at T3 were randomised into either an ad libitum or an intervention group (see below for details). Participants were excluded from the study if they had arterial hypertension, an endocrine disorder, food allergies, malabsorption, recent changes in their smoking habits, or were receiving medications that are known to interfere with energy balance. Moreover, when screening for mental problems using a standardized clinical interview revealed borderline personality disorder, substance addiction, delusional, anxiety, or affective disorders, participants were excluded. Besides, a lifetime diagnosis of a dementia, multiple sclerosis, Parkinson’s disease, stroke, epilepsy, brain tumour led to study exclusion. Finally, identification of any neurological disorder by a neuroradiologist based on MRI scans acquired during study visits led to exclusion.

**Software and computational tools**

Statistical and imaging analyses were performed using FreeSurfer (v7.4.1; RRID:SCR_001847), R (v4.4.3; RRID:SCR_001905) with the effectsize package (v1.0.0; RRID:SCR_026983), the Shapley Toolbox for Python (v0.42.1; RRID:SCR_021362), Python (v3.8.5; RRID:SCR 008394), MATLAB (R2023b; RRID:SCR_001622), SPM12 (v6225; RRID:SCR_007037) and the Computational Anatomy Toolbox CAT12 (vCAT12.8.2; RRID:SCR_019184).

**Supplementary analysis 1: Metabolic-inflammatory markers and brain-PAD - Impact of acquisition delay**

To evaluate a putative impact of the delay in acquisition between hormonal and MRI data within experimental time points for individual participants, we here again tested associations between the four hormonal measures and brain-PAD but now controlled the maximally allowed temporal delay in the acquisition of both parameters (28 or 14 days for a participant at any experimental time point).

**Supplementary analysis 2: Role of selection bias for observed treatment effects and associations**

An important question in a clinical study is whether the observed results might have been affected by selection bias – a drop-out during follow-up systematically depending on clinical variables, such as a high or low BMI or brain-PAD in our case. To address this point, we here repeated the analyses of treatment effects and of associations among BMI, metabolic parameters, brain-PAD and neuropsychological test scores exclusively based on data from those patients who did not drop-out and participated in all visits (entering a specific analysis; see below) respectively. We refer to the patients fulfilling this criterion as “completers” in the following.

Specifically, the MMS trial comprised three visits (T0, T3 and T4) and all analyses were conducted based on these three visits. Thus, given that 41 patients consistently participated in all visits (i.e., 41 completers existed), 123 data points were available for analyses constrained to “completers” in MMS. In Maintain, the situation was slightly different. Here, analyses of treatment effects and of associations between BMI and brain-PAD were conducted based on all five visits included in the protocol (T0, T3, T15, T27, T39) and analyses testing associations between BMI and metabolic parameters as well as between metabolic parameters and brain-PAD were conducted based on the first three visits (T0, T3, T15) due to the availability of metabolic parameters for only these visits. Thus, given that six participants completed all five visits, 30 data points were available for analyses of treatment effects as well as of associations between BMI and brain-PAD for Maintain. Furthermore, 16 participants existed who consistently completed T0, T3 and T15 which resulted in 48 available data points for analyses testing associations between BMI and metabolic parameters as well as between metabolic parameters and brain-PAD. After the data of completers were identified, we conducted the corresponding analyses as described in the main text.

**Supplementary analysis 3: Treatment effects and associations between BMI, metabolic parameters, brain-PAD and neuropsychological parameters across visits T0 and T3**

In order to identify potential reasons for differences between effects and associations across trials observed in some cases in the main text, we here repeated the analyses on treatment effects and on associations between BMI, metabolic parameters, brain-PAD and neuropsychological parameters but now only evaluated the data acquired at the two visits consistently included in both trials, T0 and T3. Please note that under these circumstances, evaluating effects of treatment on BMI and brain-PAD simplified to testing effects of time since diet onset ∙ group in MMS and to effects of time since diet onset in Maintain as no visits were included here that took place after diet offset except for T3.

**Supplementary analysis 4: Visit-wise associations between metabolic-inflammatory markers and brain-PAD and between brain-PAD and neuropsychological markers**

Here, we used linear models (LMs) to test associations between metabolic-inflammatory markers and brain-PAD as well between brain-PAD neuropsychological functioning separately for each visit individually to provide a rough insight into the relative contribution of cross-sectional signal covariation to the longitudinal associations focused on in this work. Specifically, to test intra-visit associations between a given metabolic-inflammatory marker (i.e., the CI) and brain-PAD (the DV), we computed the following LM for the MMS trial:

Brain-PAD = 1 + metabolic-inflammatory marker + group + age at baseline + age + BMI at baseline + presence of a metabolic syndrome + application of diabetes drugs + ventricular volume + mean Euler number

In this equation, the 1 next to the equality sign reflects the intercept of the model. All variables besides the CI and the DV were CNI. For Maintain, the LM additionally included the participants’ sex. Moreover, to test associations between brain-PAD and neuropsychological functioning in participants of the MMS trial, we evaluated this model:

Neuropsychological parameter = 1 + brain-PAD + age at baseline + age + ventricular volume + mean Euler number

As for the longitudinal analyses in the main text, we conducted directed tests and corrected for multiple testing with the FDR method. To improve comparability between the results obtained by these classical LMs and the results obtained by the LMM-based analyses conducted in the main text, data determined by the outlier exclusion procedure conducted in the LMM analyses were used for the LM analysis (instead of excluding outliers based on the same interquartile-range-based criterion individually for each visit).

Finally, to understand potential differences arising in analysing cross-sectional data with LMs and in analysing longitudinal data using LMMs, we conducted a complementary analysis. In a first step, we plotted the results for the aforementioned visit-wise LMs regressing brain-PAD onto leptin for MMS as scatter graphs (MMS and Leptin were selected for illustrative purposes). Second, we pooled the data across visits and computed a classical LM across the pooled data. Finally, for comparison, we again conducted/depicted the results for the LMM analysis of the main text regressing brain-PAD onto leptin. Across models, data determined by the outlier exclusion procedure conducted in the LMM analysis were used. The comparison between the classical LM computed for the pooled data and the LMM was conducted as these two models only differ in terms of the random intercept: the LMM does but the LM does not model this parameter (i.e., the participant-specific mean across visits). Consequently, if both models come to different results, they can be attributed to this difference.

**Supplementary analysis 5: Post hoc sensitivity analyses**

To determine the statistical power of analyses testing treatment effects as well as associations between BMI, metabolic-inflammatory markers, brain-PAD and neuropsychological parameters we here conducted post hoc sensitivity analyses with the LME_REALIZED_POWER.M algorithm provided by Bernal-Rusiel et al. (1) ( <https://github.com/NeuroStats/lme>) that determines the observed power for a given LMM. Please note that, to evaluate the impact of outlier exclusion performed for all LMMs computed in our study, we did this once based on the data were outliers were excluded and once on data where this was not the case. Please also note that for the analyses of associations between brain-PAD and neuropsychological parameters not outliers were identified and excluded at all.

**Results**

**Regional neural substrates of brain age**

Table S1 below provides detailed information on regions contributing significantly to brain age predictions of the CNN model.

**Supplementary Table S1. Regional neural substrates of brain age.**

For each trial (MMS and Maintain), voxel-wise linear mixed-effects models (LMMs) were estimated using the same specification as for testing effects of participation in the weight management programme on brain-PAD (see above) and fitted to all MRI data available for that trial. For each significant cluster (≥25 voxels; voxel-wise α_FWE = 0.025, one-sided, Bonferroni-corrected separately for positive and negative effects), the table lists the MNI peak coordinates (x, y, z), cluster size (CS), t-statistic for the fixed intercept, one-sided family-wise error–corrected p-value (p_FWE_), the intercept estimate (b), and its 95% FWE-corrected confidence interval (CI_FWE_). Positive b values indicate regions where Shapley values are on average positive (contributing to higher/older brain age estimates), and negative b values indicate regions where Shapley values are on average negative (contributing to lower/younger brain age estimates).

| **Trial / Region** | **x** | **y** | **z** | **CS** | **t** | **p_FWE_** | **b** | **CI_FWE_** |
| --- | --- | --- | --- | --- | --- | --- | --- | --- |
| **MMS (positive)** |  |  |  |  |  |  |  |  |
| Brain stem | -8 | -36 | -8 | 3∙10^4^ | 24.2 | 6∙10^-44^ | 0.056 | 0.044 – 0.068 |
| Mid. cing. gy. | -4 | 14 | 24 | 896 | 13.4 | 7∙10^-21^ | 0.017 | 0.010 – 0.024 |
| Cerebr. WM | -16 | 16 | 30 | 648 | 12.8 | 1∙10^-19^ | 0.012 | 7∙10^-3^ – 0.017 |
| Cerebr. WM | -50 | -8 | -12 | 440 | 12.6 | 4∙10^-19^ | 9∙10^-3^ | 5∙10^-3^ – 0.012 |
| Frontal pole | 4 | 70 | -10 | 1320 | 12.5 | 8∙10^-19^ | 0.011 | 7∙10^-3^ – 0.016 |
| Mid. temporal gy. | 60 | 4 | -24 | 1256 | 11.7 | 7∙10^-17^ | 8∙10^-3^ | 4∙10^-3^ – 0.012 |
| Cerebr. WM | -28 | -2 | -16 | 504 | 11.5 | 2∙10^-16^ | 6∙10^-3^ | 3∙10^-3^ – 8∙10^-3^ |
| Frontal pole | -8 | 70 | -8 | 1216 | 11.5 | 3∙10^-16^ | 0.010 | 6∙10^-3^ – 0.015 |
| Cerebr. WM | -20 | -32 | 42 | 576 | 11.2 | 1∙10^-15^ | 6∙10^-3^ | 3∙10^-3^ – 9∙10^-3^ |
| Ventral dienceph. | -12 | -22 | -12 | 424 | 10.8 | 1∙10^-14^ | 0.022 | 0.011 – 0.033 |
| Cerebr. WM | 14 | 26 | 28 | 624 | 10.5 | 6∙10^-14^ | 6∙10^-3^ | 3∙10^-3^ – 8∙10^-3^ |
| Precentral gy. | 60 | 12 | 26 | 312 | 10.4 | 1∙10^-13^ | 0.016 | 8∙10^-3^ – 0.024 |
| Planum polare | 44 | -12 | -6 | 880 | 10.2 | 4∙10^-13^ | 4∙10^-3^ | 2∙10^-3^ – 6∙10^-3^ |
| Cerebr. WM | 18 | -32 | 40 | 592 | 10.1 | 7∙10^-13^ | 7∙10^-3^ | 3∙10^-3^ – 0.010 |
| Postcentral gy. | 44 | -12 | 38 | 1016 | 9.9 | 1∙10^-12^ | 0.016 | 7∙10^-3^ – 0.024 |
| Posterior insula | 36 | 4 | -16 | 520 | 9.9 | 2∙10^-12^ | 5∙10^-3^ | 2∙10^-3^ – 7∙10^-3^ |
| Precentral gy. | -62 | 8 | 32 | 616 | 9.9 | 2∙10^-12^ | 0.025 | 0.011 – 0.038 |
| Cerebr. WM | 16 | -52 | 36 | 568 | 9.9 | 2∙10^-12^ | 4∙10^-3^ | 2∙10^-3^ – 5∙10^-3^ |
| Posterior insula | -44 | -6 | -6 | 360 | 9.7 | 7∙10^-12^ | 5∙10^-3^ | 2∙10^-3^ – 8∙10^-3^ |
| Postcentral gy. | -46 | -18 | 42 | 248 | 9.6 | 1∙10^-11^ | 6∙10^-3^ | 3∙10^-3^ – 0.010 |
| Temporal pole | -52 | 20 | -24 | 456 | 9.5 | 1∙10^-11^ | 0.014 | 6∙10^-3^ – 0.022 |
| Postcentral gy. | 66 | -6 | 16 | 224 | 9.3 | 5∙10^-11^ | 7∙10^-3^ | 3∙10^-3^ – 0.011 |
| Mid. temporal gy. | -68 | -6 | -18 | 272 | 9.1 | 2∙10^-10^ | 0.016 | 7∙10^-3^ – 0.026 |
| Med. frontal ctx. | 0 | 42 | -20 | 336 | 8.8 | 7∙10^-10^ | 6∙10^-3^ | 3∙10^-3^ – 0.010 |
| Cerebr. WM | -8 | -54 | -20 | 248 | 8.7 | 1∙10^-9^ | 8∙10^-3^ | 3∙10^-3^ – 0.013 |
| Cerebr. WM | 28 | 8 | 28 | 224 | 8.6 | 2∙10^-9^ | 9∙10^-3^ | 3∙10^-3^ – 0.014 |
| Mid. frontal gy. | -50 | 46 | 14 | 344 | 8.5 | 4∙10^-9^ | 8∙10^-3^ | 3∙10^-3^ – 0.013 |
| Sup. frontal gy. | -28 | 64 | 18 | 264 | 8.4 | 6∙10^-9^ | 0.010 | 4∙10^-3^ – 0.017 |
| Inf. frontal gy. op. pt. | -60 | 22 | 18 | 272 | 8.3 | 1∙10^-8^ | 8∙10^-3^ | 3∙10^-3^ – 0.013 |
| Anterior cing. gy. | -4 | 24 | 24 | 248 | 7.9 | 9∙10^-8^ | 9∙10^-3^ | 3∙10^-3^ – 0.015 |
| Mid. frontal gy. | 36 | 62 | 6 | 216 | 7.5 | 7∙10^-7^ | 5∙10^-3^ | 1∙10^-3^ – 8∙10^-3^ |
|  |  |  |  |  |  |  |  |  |
| **MMS (negative)** |  |  |  |  |  |  |  |  |
| Thalamus proper | -10 | -34 | -2 | 1032 | -21.5 | 8∙10^-39^ | -0.028 | -0.035 - -0.021 |
| Brain stem | -2 | -32 | -8 | 8408 | -20.2 | 3∙10^-36^ | -0.039 | -0.049 - -0.029 |
| Med. orbital gy. | -14 | 28 | -22 | 544 | -16.8 | 8∙10^-29^ | -0.011 | -0.014 - -7∙10^-3^ |
| Brain stem | -6 | -34 | -10 | 544 | -16.1 | 3∙10^-27^ | -0.022 | -0.030 - -0.015 |
| Putamen | 24 | 14 | 2 | 752 | -16.0 | 5∙10^-27^ | -7∙10^-3^ | -9∙10^-3^ - -5∙10^-3^ |
| Mid. cing. gy. | 4 | 0 | 30 | 280 | -15.5 | 6∙10^-26^ | -0.030 | -0.040 - -0.020 |
| Mid. cing. gy. | -2 | -2 | 30 | 656 | -15.5 | 6∙10^-26^ | -0.021 | -0.028 - -0.014 |
| Cerebr. WM | -22 | 16 | -10 | 576 | -14.9 | 2∙10^-24^ | -0.010 | -0.014 - -7∙10^-3^ |
| Lat. ventricle | -18 | -40 | 12 | 1128 | -14.4 | 2∙10^-23^ | -0.010 | -0.014 - -7∙10^-3^ |
| Caudate | -14 | 16 | 6 | 304 | -14.2 | 7∙10^-23^ | -0.013 | -0.017 - -8∙10^-3^ |
| Thalamus proper | -8 | -24 | 2 | 344 | -13.6 | 2∙10^-21^ | -0.024 | -0.033 - -0.015 |
| Cerebr. WM | 10 | 26 | 12 | 416 | -13.1 | 3∙10^-20^ | -0.016 | -0.022 - -9∙10^-3^ |
| Med. Orbital gy. | 8 | 44 | -22 | 640 | -13.0 | 5∙10^-20^ | -0.011 | -0.015 - -6∙10^-3^ |
| Planum polare | -42 | 4 | -18 | 616 | -12.3 | 2∙10^-18^ | -2∙10^-3^ | -4∙10^-3^ - -1∙10^-3^ |
| Planum polare | 40 | -12 | -8 | 520 | -11.3 | 6∙10^-16^ | -0.010 | -0.015 - -5∙10^-3^ |
| Thalamus proper | 10 | -32 | 2 | 568 | -11.2 | 1∙10^-15^ | -0.025 | -0.036 - -0.013 |
| Cerebr. WM | -30 | -36 | 14 | 544 | -10.6 | 4∙10^-14^ | -0.011 | -0.016 - -5∙10^-3^ |
| Cerebr. WM | -14 | 28 | 10 | 432 | -10.5 | 5∙10^-14^ | -9∙10^-3^ | -0.014 - -5∙10^-3^ |
| Postcentral gy. | 46 | -14 | 40 | 256 | -9.8 | 4∙10^-12^ | -7∙10^-3^ | -0.011 - -3∙10^-3^ |
| Cerebr. WM | 26 | -30 | 20 | 200 | -9.6 | 7∙10^-12^ | -4∙10^-3^ | -6∙10^-3^ - -2∙10^-3^ |
| Cerebr. WM | -24 | -34 | 38 | 304 | -9.0 | 3∙10^-10^ | -4∙10^-3^ | -6∙10^-3^ - -2∙10^-3^ |
| Cerebr. WM | -26 | 28 | 4 | 208 | -8.9 | 6∙10^-10^ | -6∙10^-3^ | -0.010 - -3∙10^-3^ |
| Thalamus proper | -16 | -14 | 12 | 272 | -8.9 | 6∙10^-10^ | -0.016 | -0.025 - -6∙10^-3^ |
| Parahipp. gy. | -24 | -26 | -26 | 200 | -7.9 | 9∙10^-8^ | -5∙10^-3^ | -8∙10^-3^ - -2∙10^-3^ |
| Med. frontal ctx. | -2 | 34 | -24 | 344 | -7.9 | 1∙10^-7^ | -0.012 | -0.021 - -4∙10^-3^ |
|  |  |  |  |  |  |  |  |  |
| **Maintain (positive)** |  |  |  |  |  |  |  |  |
| Caudate | -20 | -20 | 22 | 7352 | 18.3 | 9∙10^-27^ | 0.015 | 0.010-0.019 |
| Brain stem | 6 | -34 | -8 | 2704 | 16.8 | 3∙10^-24^ | 0.022 | 0.015-0.029 |
| Lat. ventricle | 18 | -24 | 22 | 2376 | 16.0 | 8∙10^-23^ | 0.026 | 0.018-0.035 |
| Ventral dienceph. | 2 | -16 | -16 | 560 | 13.2 | 1∙10^-17^ | 0.028 | 0.017-0.039 |
| Cerebr. WM | -36 | -58 | 2 | 360 | 12.2 | 1∙10^-15^ | 6∙10^-3^ | 3∙10^-3^-7∙10^-3^ |
| Cerebr. WM | -20 | -26 | 40 | 392 | 12.0 | 3∙10^-15^ | 5∙10^-3^ | 3∙10^-3^-7∙10^-3^ |
| Cerebr. WM | 26 | 12 | 6 | 200 | 11.6 | 2∙10^-14^ | 7∙10^-3^ | 4∙10^-3^-0.105 |
| Mid. cing. gy. | 2 | -14 | 32 | 504 | 10.5 | 4∙10^-12^ | 0.010 | 5∙10^-3^-0.016 |
|  |  |  |  |  |  |  |  |  |
| **Maintain (negative)** |  |  |  |  |  |  |  |  |
| Ventral dienceph. | -4 | -24 | -2 | 944 | -14.3 | 9∙10^-20^ | -8∙10^-3^ | -0.010 - -5∙10^-3^ |
| Mid. cing. gy. | -2 | -4 | 30 | 272 | -14.0 | 4∙10^-19^ | -0.013 | -0.018 - -8∙10^-3^ |
| Parahipp. gy. | -14 | -32 | -8 | 336 | -14.0 | 4∙10^-19^ | -0.015 | -0.020 - -9∙10^-3^ |
| Brain stem | 6 | -36 | -6 | 328 | -13.9 | 7∙10^-19^ | -0.010 | -0.014 - -6∙10^-3^ |
| Lat. ventricle | -26 | -38 | 14 | 728 | -13.7 | 2∙10^-18^ | -5∙10^-3^ | -6∙10^-3^ - -3∙10^-3^ |
| Lat. ventricle | 4 | 18 | -2 | 512 | -12.5 | 2∙10^-16^ | -9∙10^-3^ | -0.013 - -5∙10^-3^ |
| Lat. ventricle | 18 | -30 | 16 | 472 | -12.2 | 9∙10^-16^ | -0.010 | -0.015 - -6∙10^-3^ |
| Cerebr. WM | -8 | 0 | 6 | 672 | -12.0 | 2∙10^-15^ | -0.020 | -0.029 - -0.011 |
| Cerebr. WM | 18 | -42 | 14 | 256 | -10.6 | 2∙10^-12^ | -7∙10^-3^ | -0.010 - -3∙10^-3^ |
| Brain stem | -6 | -36 | -12 | 232 | -10.5 | 3∙10^-12^ | -0.010 | -0.015 - -5∙10^-3^ |
| Putamen | 24 | 12 | -6 | 272 | -9.2 | 1∙10^-09^ | -0.010 | -0.015 - -4∙10^-3^ |

Abbreviations: Cerebr., cerebral; cing., cingulate; ctx., cortex; dienceph., diencephalon; gy., gyrus; lat., lateral; med., medial; mid., middle; op., opercular; parahipp., parahippocampal; pt., part; sup., superior; WM, white matter; CS, cluster size (mm³); p_FWE, one-sided family-wise error–corrected p-value; CI_FWE, 95% family-wise error–corrected confidence interval; x, y, z, MNI coordinates of voxel with peak effect.

**Supplementary analysis 1: Hormonal functioning and brain-PAD - Impact of acquisition delay**

Compared to the analyses in the main text, the basic pattern of associations appeared nearly unchanged except for the fact that the associations between hormonal markers and brain-PAD failed to reach significance for the shortest time delay of 14 days in Maintain (for which only a limited amount of data was available). Fig. S1 below summarizes the results.


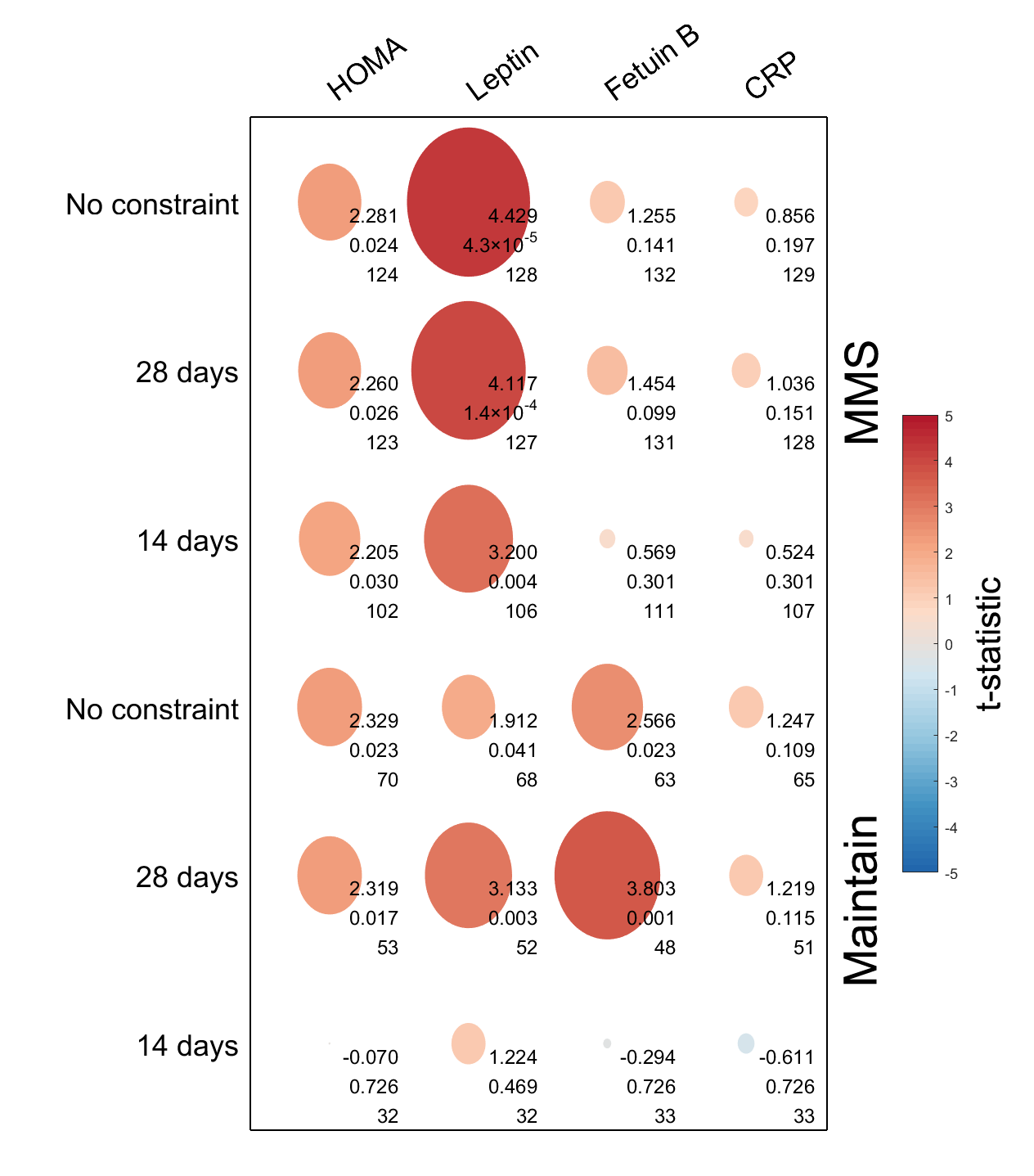


**Figure S1. Associations between metabolic-inflammatory markers and brain-PAD for different maximal time delays in acquisition of both parameters.**

The figure shows the links between the four metabolic-inflammatory markers and brain-PAD separately for the two data sets and three acquisition delay constraints. Specifically, the first and fourth row again depict the results shown in the main text as reference which did not apply an acquisition delay constraint. The second and fifth rows depict the results obtained for those data for which the delay in acquisition between the metabolic-inflammatory and MRI (i.e., brain-PAD) measures was 28 days at maximum. The third and sixth rows show results for maximal delay constraint of 14 days. Numbers in each bubble indicate, from top to bottom, the t-statistic, the corresponding FDR-corrected p-value, and the number of observations included in that analysis. Statistical models and covariate adjustment are identical to those used in the main analyses.

**Supplementary analysis 2: Role of selection bias for observed treatment effects and associations**

Supplementary table S2 below reports the results obtained in analyses testing putative effects of selection bias. To promote comparability, the results obtained in a given analysis for all available data points reported in the main text are depicted first and then the results obtained for the corresponding analysis based on data of completers only are depicted.

**Supplementary Table S2: Role of selection bias for observed treatment effects and associations**

The table contrasts results from linear mixed-effects models fitted to (1.) treatment effects on BMI and brain-predicted age difference (brain-PAD) and their association over time in both trials (MMS and Maintain), (2.) associations between BMI and metabolic-inflammatory markers (HOMA-IR, leptin, fetuin-B, C-reactive protein [CRP]) across time, (3.) associations between metabolic-inflammatory markers and brain-PAD across time, and (4.) associations between brain-PAD and neuropsychological measures across time in the MMS trial only. For each analysis, rows labelled “All” refer to models using all available observations, whereas rows labelled “Completers” refer to models restricted to participants who completed all planned visits. Entries report t-statistics (t) and corresponding p-values (p or pFDR, where pFDR denotes p-values adjusted for multiple testing using the false discovery rate).

| 1. Effect of treatment on BMI and brain-PAD and association of BMI and brain-PAD across time | | | | | | | | | | |
| --- | --- | --- | --- | --- | --- | --- | --- | --- | --- | --- |
| MMS | BMI | | BMI | | BPAD | | | BPAD | |  |
|  | Time since diet onset ∙ group | | Time since diet offset ∙ group | | Time since diet onset ∙ group | | | Time since diet offset ∙ group | | Association BMI and brain-PAD |
| All | t=-6.52 p=7.5∙10^-10^ | | t=1.36 p=0.176 | | t=-2.02 p=0.023 | | | t=1.07 p=0.285 | | t=4.80 p=2.2∙10^-6^ |
| Completers | t=-6.37 p=2.1∙10^-9^ | | t=1.34 p=0.184 | | t=-1.95 p=0.027 | | | t=1.04 p=0.301 | | t=4.82 p=2.3∙10^-6^ |
| Maintain |  | |  | |  | | |  | |  |
|  | Time since diet onset | | Time since diet offset ∙ group | | Time since diet onset | | | Time since diet offset ∙ group | | Association BMI and brain-PAD |
| All | t=-6.82 p=4.9∙10^-10^ | | t=-0.12 p=0.901 | | t=-7.37 p=4.2∙10^-11^ | | | t=-0.81 p=0.420 | | t=2.52 p=0.007 |
| Completers | t=-4.73 p=5.2∙10^-5^ | | t=2.77 p=0.112 | | t=-4.19 p=2.3∙10^-4^ | | | t=-1.94 p=0.066 | | t=0.88 p=0.195 |
| 2. Associations between BMI and parameters of metabolic inflammation across time | | | | | | | | | | |
| MMS |  | | |  | |  | | |  | |
|  | HOMA-IR | | | Leptin | | Fetuin B | | | CRP | |
| All | t=3.92 p_FDR_=9.9∙10^-5^ | | | t=10.97 p_FDR_=1.3∙10^-19^ | | t=4.08 p_FDR_=7.9∙10^-5^ | | | t=3.11  p_FDR_=0.001 | |
| Completers | t=3.53 p_FDR_=4.0∙10^-4^ | | | t=10.77 p_FDR_=1.5∙10^-18^ | | t=4.41 p_FDR_=2.4∙10^-5^ | | | t=2.86 p_FDR_=0.003 | |
| Maintain |  | | |  | |  | | |  | |
|  | HOMA-IR | | | Leptin | | Fetuin B | | | CRP | |
| All | t=3.32 p_FDR_=0.002 | | | t=7.49 p_FDR_=8.9∙10^-10^ | | t=0.41 p_FDR_=0.347 | | | t=1.33 p_FDR_=0.126 | |
| Completers | t=4.01 p_FDR_=2.9∙10^-4^ | | | t=6.88 p_FDR_=8.3∙10^-8^ | | t=0.95 p_FDR_=0.231 | | | t=0.63 p_FDR_=0.268 | |
| 3. Associations between parameters of metabolic inflammation and brain-PAD across time | | | | | | | | | | |
| MMS |  | | |  | |  | | |  | |
|  | HOMA-IR | | | Leptin | | Fetuin B | | | CRP | |
| All | t=2.28 p_FDR_=0.024 | | | t=4.43 p_FDR_=4.3∙10^-5^ | | t=1.26 p_FDR_=0.141 | | | t=0.86 p_FDR_=0.197 | |
| Completers | t=2.38 p_FDR_=0.019 | | | t=4.59 p_FDR_=2.5∙10^-5^ | | t=1.30 p_FDR_=0.130 | | | t=0.78 p_FDR_=0.220 | |
| Maintain |  | | |  | |  | | |  | |
|  | HOMA-IR | | | Leptin | | Fetuin B | | | CRP | |
| All | t=2.33 p_FDR_=0.023 | | | t=1.91 p_FDR_=0.041 | | t=2.57 p_FDR_=0.023 | | | t=1.25 p_FDR_=0.109 | |
| Completers | t=2.47 p_FDR_=0.025 | | | t=1.00 p_FDR_=0.161 | | t=2.35 p_FDR_=0.025 | | | t=1.50 p_FDR_=0.095 | |
| 4. Associations between brain-PAD and neuropsychological measures across time in the MMS trial | | | | | | | | | | |
|  | | All | | | | | Completers | | | |
| Digit span | | t = -1.50, p = 0.069 | | | | | t = -1.46, p = 0.074 | | | |
| Verbal fluency, phon. | | t = 0.20, p_FDR_ = 0.580 | | | | | t = 0.40, p_FDR_ = 0.655 | | | |
| Verbal fluency, sem. | | t = -1.31, p_FDR_ = 0.193 | | | | | t = -1.10, p_FDR_ = 0.281 | | | |
| Trail making test, pt. A | | t = 2.32, p_FDR_ = 0.022 | | | | | t = 2.23, p_FDR_ = 0.028 | | | |
| Trail making test, pt. B | | t = 0.29, p_FDR_ = 0.387 | | | | | t = 0.36, p_FDR_ = 0.359 | | | |
| VLMT: recognition | | t = -0.52, p_FDR_ = 0.302 | | | | | t = -0.43, p_FDR_ = 0.335 | | | |
| VLMT: learning | | t = -0.56, p_FDR_ = 0.302 | | | | | t = -0.50, p_FDR_ = 0.335 | | | |
| VLMT: delayed recall | | t = -1.18, p_FDR_ =0.302 | | | | | t = -1.17, p_FDR_ = 0.335 | | | |
| VLMT: consolidation | | t = 0.87, p_FDR_ = 0.302 | | | | | t = 0.92, p_FDR_ = 0.335 | | | |
| Stroop colour-word interf. | | t = 0.05, p = 0.481 | | | | | t = -0.15, p = 0.558 | | | |

Note: Overall, the pattern of results shows only minor differences between analyses based on all available data and those based on completers only, suggesting that selection bias due to attrition is unlikely to account for the observed treatment effects and associations.

**Supplementary analysis 3: Treatment effects and associations between BMI, metabolic parameters, brain-PAD and neuropsychological parameters across visits T0 and T3**

Supplementary table S3 below present the results obtained for analyses conducted based on data only acquired at T0 and T3. To promote comparability, the results obtained for a given longitudinal LMM analysis covering the whole trial period (i.e., the results reported in the main text) are reported first and then the results obtained for the data constrained to visits T0 and T3 are reported.

**Supplementary Table S3: Effect of treatment on BMI and brain-PAD and association of BMI and brain-PAD across time**

The table summarises results from longitudinal LMMs assessing (1.) the effect of treatment on BMI and brain-predicted age difference (brain-PAD) and the association between BMI and brain-PAD over time, (2.) associations between BMI and metabolic-inflammatory markers (HOMA-IR, leptin, fetuin-B, CRP) across time, and (3.) associations between metabolic-inflammatory markers and brain-PAD across time in the MMS and Maintain trials. For each model, rows labelled “Full period” show estimates based on all available visits, whereas rows labelled “T0 & T3 only” show the corresponding estimates when restricting the data to baseline (T0) and end-of-intervention (T3) visits. Entries report t-statistics (t) and p-values (p for unadjusted tests, pFDR for p-values adjusted for multiple testing using the false discovery rate).

| 1. Effect of treatment on BMI and brain-PAD and association of BMI and brain-PAD across time | | | | | | | | | |
| --- | --- | --- | --- | --- | --- | --- | --- | --- | --- |
| Full period | BMI | | BMI | | Brain-PAD | | Brain-PAD | |  |
|  | Time since diet onset ∙ group | | Time since diet offset ∙ group | | Time since diet onset ∙ group | | Time since diet offset ∙ group | | Association BMI and brain-PAD |
| MMS | t=-6.52 p=7.5∙10^-10^ | | t=1.36 p=0.176 | | t=-2.02 p=0.023 | | t=1.07 p=0.285 | | t=4.80 p=2.2∙10^-6^ |
| Maintain | t=-6.82 p=4.9∙10^-10^ | | t=-0.12 p=0.901 | | t=-7.37 p=4.2∙10^-11^ | | t=-0.81 p=0.420 | | t=2.52 p=0.007 |
| T0 & T3 only |  | |  | |  | |  | |  |
| MMS | t=-11.29 p=5.1∙10^-19^ | |  | | t=-3.54 p=3.2∙10^-4^ | |  | | t=4.35 p=1.9∙10^-5^ |
| Maintain | t=-10.53 p=1.2∙10^-14^ | |  | | t=-3.96 p=1.5∙10^-4^ | |  | | t=0.04 p=0.969 |
| 2. Associations between BMI and parameters of metabolic inflammation across time | | | | | | | | | |
| Full period | |  | |  | |  | |  | |
|  | | HOMA-IR | | Leptin | | Fetuin B | | CRP | |
| MMS | | t=3.92 p_FDR_=9.9∙10^-5^ | | t=10.97 p_FDR_=1.3∙10^-19^ | | t=4.08 p_FDR_=7.9∙10^-5^ | | t=3.11 p_FDR_=0.001 | |
| Maintain | | t=3.32 p_FDR_=0.002 | | t=7.49 p_FDR_=8.9∙10^-10^ | | t=0.41 p_FDR_=0.347 | | t=1.33 p_FDR_=0.126 | |
| T0 & T3 only | |  | |  | |  | |  | |
|  | | HOMA-IR | | Leptin | | Fetuin B | | CRP | |
| MMS | | t=3.69 p_FDR_=2.7∙10^-4^ | | t=7.50 p_FDR_=1.3∙10^-10^ | | t=4.64 p_FDR_=1.3∙10^-5^ | | t=2.37 p_FDR_=0.010 | |
| Maintain | | t=1.68 p_FDR_=0.102 | | t=6.64 p_FDR_=1.2∙10^-7^ | | t=0.43 p_FDR_=0.445 | | t=-0.18 p_FDR_=0.573 | |
| 3. Associations between parameters of metabolic inflammation and brain-PAD across time | | | | | | | | | |
| Full period | |  | |  | |  | |  | |
|  | | HOMA-IR | | Leptin | | Fetuin B | | CRP | |
| MMS | | t=2.28 p_FDR_=0.024 | | t=4.43 p_FDR_=4.3∙10^-5^ | | t=1.26 p_FDR_=0.141 | | t=0.86 p_FDR_=0.197 | |
| Maintain | | t=2.33 p_FDR_=0.023 | | t=1.91 p_FDR_=0.041 | | t=2.57 p_FDR_=0.023 | | t=1.25 p_FDR_=0.109 | |
| T0 & T3 only | |  | |  | |  | |  | |
|  | | HOMA-IR | | Leptin | | Fetuin B | | CRP | |
| MMS | | t=1.53 p_FDR_=0.087 | | t=4.39 p_FDR_=6.8∙10^-5^ | | t=2.02 p_FDR_=0.046 | | t=-0.37 p_FDR_=0.645 | |
| Maintain | | t=1.42 p_FDR_=0.163 | | t=0.987 p_FDR_=0.2203 | | t=2.22 p_FDR_=0.066 | | t=0.75 p_FDR_=0.228 | |

Note: Overall, the pattern of treatment effects and associations is broadly comparable between the full-period analyses and the T0/T3-restricted analyses, with some attenuation of effects in the more constrained models, particularly in the Maintain trial.

**Supplementary analysis 4: Visit-wise associations between** **metabolic-inflammatory markers and brain-PAD and between brain-PAD and neuropsychological markers**

Panels a – c of Figure S2 summarize the results obtained for testing the mentioned associations visit by visit, panel d depicts the results of the complementary analysis comparing visit-wise linear models (LMs) with a linear mixed-effects model (LMMs).


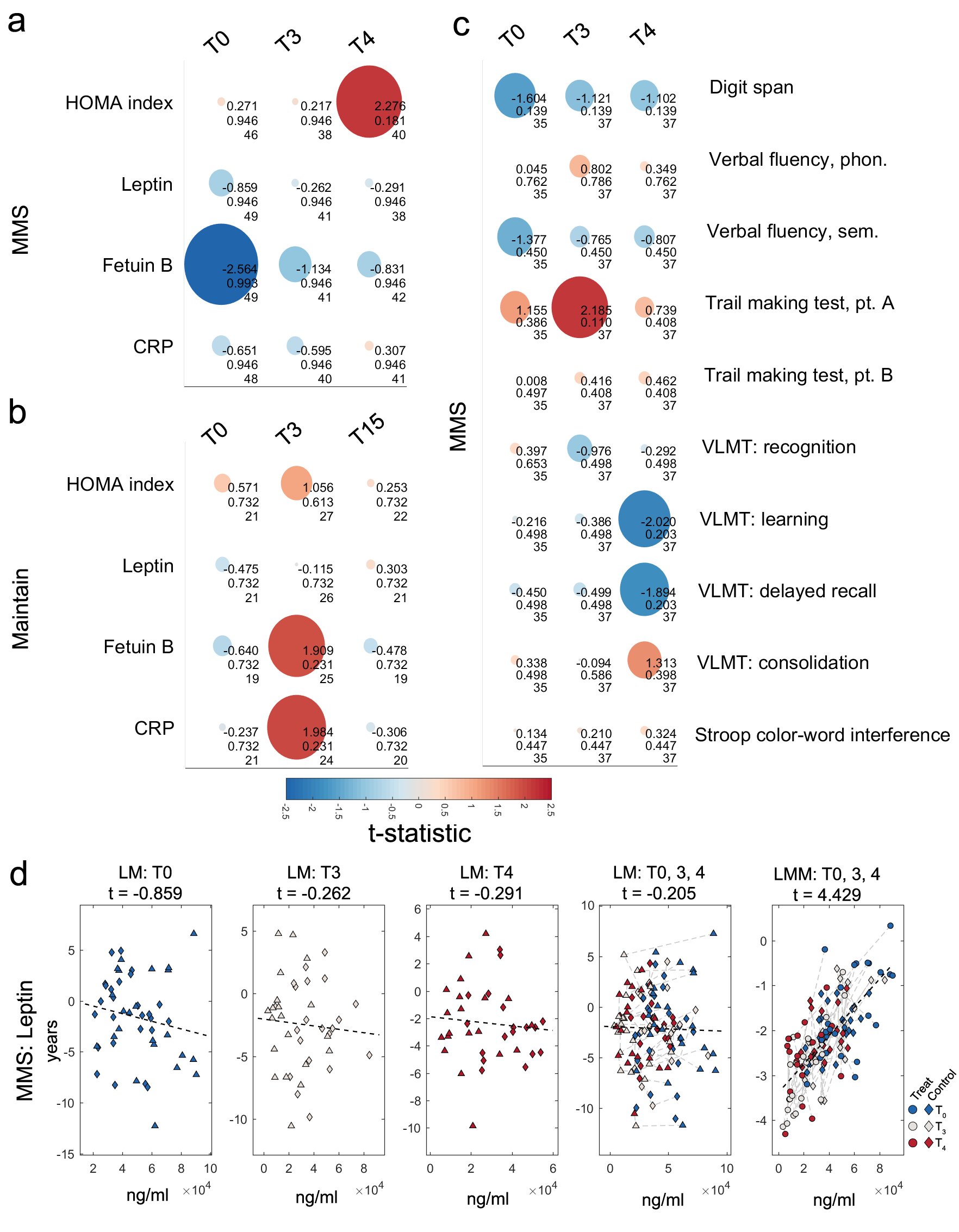


**Figure S2. Visit-wise associations between metabolic-inflammatory markers and brain-PAD and between brain-PAD and neuropsychological functioning.**

Panels a–c display visit-wise associations, and panel d shows results from the complementary analysis.

(a) Associations between metabolic-inflammatory markers and brain-predicted age difference (brain-PAD) in the MMS trial.

(b) Corresponding associations for the Maintain trial.

(c) Associations between brain-PAD and neuropsychological measures at each visit.

In panels a–c, the numbers within each circle (from top to bottom) denote the t-statistic, the one-sided p-value corrected for multiple testing using the false discovery rate (pFDR), and the number of evaluated observations. For metabolic-inflammatory markers in MMS and Maintain, the FDR procedure was applied across 12 tests per trial (four markers × three visits). For cognitive measures, the number of tests entering the FDR correction was given by the number of outcome measures within an instrument multiplied by the number of visits (e.g. VLMT: four measures × three visits = 12 tests; verbal fluency: two measures × three visits = six tests, etc.).

(d) Results of the complementary analysis. From left to right, the panel shows three separate visit-wise LMs regressing brain-PAD on leptin, a LM fitted to data pooled across visits, and a LMM fitted to the same pooled data. In all scatter plots, brain-PAD values are depicted after adjustment for covariates of no interest (CNI). In the four LMs, CNI were included only as fixed effects (no random intercept), whereas the LMM additionally included a participant-level random intercept. In the LMM, leptin shows a clear positive association with brain-PAD across visits, whereas the visit-wise and pooled LMs yield numerically negative, non-significant associations. As the only difference between the pooled LM and the LMM is the inclusion of the random intercept, this comparison illustrates that the improved detection of the leptin–brain-PAD association in the LMM arises from its ability to account for between-subject variability via random effects.

**Supplementary analysis 5: Post hoc sensitivity analyses**

The results of our sensitivity analyses are depicted in the following supplementary table.

**Supplementary Table S4: Sensitivity analyses**

The table summarises sensitivity analyses assessing the robustness of (1.) treatment effects on BMI and brain-predicted age difference (brain-PAD) and the association between BMI and brain-PAD over time in the MMS and Maintain trials, (2.) associations between BMI and metabolic-inflammatory markers (HOMA-IR, leptin, fetuin-B, CRP), (3.) associations between metabolic-inflammatory markers and brain-PAD, and (4.) associations between brain-PAD and neuropsychological measures. For each effect, the table reports the corresponding p- or FDR-adjusted p-values (as in the main analyses) obtained from models with outlier exclusion and from models including all observations. The close similarity of values with and without outlier exclusion, and the unchanged pattern of statistically significant versus non-significant results, indicate that the main findings are robust to the handling of outliers.

| 1. Sensitivity analyses for treatment effects on BMI and brain-PAD and for association between BMI and brain-PAD | | | | | | | | | | | |
| --- | --- | --- | --- | --- | --- | --- | --- | --- | --- | --- | --- |
| MMS |  | | |  | |  | | |  | |  |
|  | BMI | | | BMI | | Brain-PAD | | | brain-PAD | |  |
|  | Time since diet onset ∙ group | | | Time since diet offset ∙ group | | Time since diet onset ∙ group | | | Time since diet offset ∙ group | | Association BMI and brain-PAD |
| With outlier  exclusion | 1.0000 | | | 0.2543 | | 0.4821 | | | 0.1741 | | 0.9959 |
| Without outlier exclusion | 1.0000 | | | 0.2543 | | 0.4821 | | | 0.1741 | | 0.9989 |
| Maintain |  | | |  | |  | | |  | |  |
|  | BMI | | | BMI | | Brain-PAD | | | brain-PAD | |  |
| With outlier exclusion | 1.0000 | | | 0.0516 | | 1.0000 | | | 0.1214 | | 0.6803 |
| Without outlier exclusion | 1.0000 | | | 0.0516 | | 0.9165 | | | 0.0565 | | 0.6630 |
| 2. Sensitivity analyses for associations between BMI and metabolic-inflammatory markers | | | | | | | | | | | |
| MMS | |  | | |  | |  | | |  | |
|  | | HOMA-IR | | | Leptin | | Fetuin B | | | CRP | |
| With outlier exclusion | | 0.9686 | | | 1.0000 | | 0.9767 | | | 0.8263 | |
| Without outlier exclusion | | 0.9872 | | | 1.0000 | | 0.9522 | | | 0.8173 | |
| Maintain | |  | | |  | |  | | |  | |
|  | | HOMA-IR | | | Leptin | | Fetuin B | | | CRP | |
| With outlier exclusion | | 0.8754 | | | 1.0000 | | 0.0746 | | | 0.2329 | |
| Without outlier exclusion | | 0.9213 | | | 1.0000 | | 0.0990 | | | 0.2897 | |
| 3. Sensitivity analyses for associations of metabolic-inflammatory markers and brain-PAD | | | | | | | | | | | |
| MMS | |  | | |  | |  | | |  | |
|  | | HOMA-IR | | | Leptin | | Fetuin B | | | CRP | |
| With outlier exclusion | | 0.5818 | | | 0.9885 | | 0.2363 | | | 0.1302 | |
| Without outlier exclusion | | 0.5719 | | | 0.9838 | | 0.2695 | | | 0.0946 | |
| Maintain | |  | | |  | |  | | |  | |
|  | | HOMA-IR | | | Leptin | | Fetuin B | | | CRP | |
| With outlier exclusion | | 0.5571 | | | 0.4242 | | 0.6320 | | | 0.1866 | |
| Without outlier exclusion | | 0.7853 | | | 0.1331 | | 0.5359 | | | 0.5198 | |
| 4. Sensitivity analyses for associations of brain-PAD and neuropsychological measures | | | | | | | | | | | |
|  | | | With outlier exclusion | | | | | Without outlier exclusion | | | |
| Digit span | | | 0.293 | | | | | 0.293 | | | |
| Verbal fluency, phon. | | | 0.054 | | | | | 0.054 | | | |
| Verbal fluency, sem. | | | 0.235 | | | | | 0.235 | | | |
| Trail making test, pt. A | | | 0.583 | | | | | 0.583 | | | |
| Trail making test, pt. B | | | 0.057 | | | | | 0.057 | | | |
| VLMT: recognition | | | 0.082 | | | | | 0.082 | | | |
| VLMT: learning | | | 0.077 | | | | | 0.077 | | | |
| VLMT: delayed recall | | | 0.195 | | | | | 0.195 | | | |
| VLMT: consolidation | | | 0.128 | | | | | 0.128 | | | |
| Stroop colour-word interf. | | | 0.050 | | | | | 0.050 | | | |

**References**

1. Bernal-Rusiel JL, Greve DN, Reuter M, Fischl B, Sabuncu MR, Initiative AsDN. Statistical analysis of longitudinal neuroimage data with linear mixed effects models. Neuroimage. 2013;66:249–60.
